# An L-type calcium channel blocker nimodipine exerts anti-fibrotic effects by attenuating TGF-β1 induced calcium response in an in vitro model of thyroid eye disease

**DOI:** 10.1186/s40662-024-00401-5

**Published:** 2024-09-06

**Authors:** Qian Chen, Yuan Pan, Yunwei Hu, Guanyu Chen, Xiaoqing Chen, Yanyan Xie, Minzhen Wang, Zhuang Li, Jun Huang, Yuxun Shi, Haixiang Huang, Te Zhang, Mei Wang, Peng Zeng, Sha Wang, Rongxin Chen, Yongxin Zheng, Liuxueying Zhong, Huasheng Yang, Dan Liang

**Affiliations:** 1 State Key Laboratory of Ophthalmology, Zhongshan Ophthalmic Center, Sun Yat-sen University, Guangdong Provincial Key Laboratory of Ophthalmology Visual Science, Guangzhou, 510060 China; 2 Department of Ophthalmology, The Third Affiliated Hospital, Sun Yat-sen University, Guangzhou, 510630 China; 3 Ophthalmic Center, The Second Affiliated Hospital, Jiangxi Medical College, Nanchang University, Nanchang, 330006 China; 4 Department of Ophthalmology, Sun Yat-sen Memorial Hospital, Sun Yat-sen University, Guangzhou, 510120 China; 5 Eye Center of Xiangya Hospital, Central South University, Hunan Key Laboratory of Ophthalmology, Changsha, 410008 China

**Keywords:** Thyroid eye disease, Fibrosis, Nimodipine, Calcium signaling, Orbital fibroblasts

## Abstract

**Background:**

Thyroid eye disease (TED) is a vision-threatening autoimmune disorder. Orbital tissue fibrosis leading to intractable complications remains a troublesome issue in TED management. Exploration of novel therapeutic targets and agents to ameliorate tissue fibrosis is crucial for TED. Recent work suggests that Ca^2+^ signaling participates in tissue fibrosis. However, whether an alteration of Ca^2+^ signaling has a role in fibrogenesis during TED remains unclear. In this study, we aimed to investigate the role of Ca^2+^ signaling in the fibrogenesis process during TED and the potential therapeutic effects of a highly selective inhibitor of the L-type calcium channel (LTCC), nimodipine, through a TGF-β1 induced in vitro TED model.

**Methods:**

Primary culture of orbital fibroblasts (OFs) were established from orbital adipose connective tissues of patients with TED and healthy control donors. Real-time quantitative polymerase chain reaction (RT-qPCR) and RNA sequencing were used to assess the genes expression associated with LTCC in OFs. Flow cytometry, RT-qPCR, 5-ethynyl-2′-deoxyuridine (EdU) proliferation assay, wound healing assay and Western blot (WB) were used to assess the intracellular Ca^2+^ response on TGF-β1 stimulation, and to evaluate the potential therapeutic effects of nimodipine in the TGF-β1 induced in vitro TED model. The roles of Ca^2+^/calmodulin-dependent protein kinase II (CaMKII) and signal transducer and activator of transcription 1 (STAT1) in fibrogenesis during TED were determined by immunohistochemistry, WB, flow cytometry and co-immunoprecipitation assay. Selective inhibitors were used to explore the downstream signaling pathways.

**Results:**

LTCC inhibitor nimodipine blocked the TGF-β1 induced intracellular Ca^2+^ response and further reduced the expression of alpha-smooth muscle actin (α-SMA), collagen type I alpha 1 (Col1A1) and collagen type I alpha 2 (Col1A2) in OFs. Besides, nimodipine inhibited cell proliferation and migration of OFs. Moreover, our results provided evidence that activation of the CaMKII/STAT1 signaling pathway was involved in fibrogenesis during TED, and nimodipine inhibited the pro-fibrotic functions of OFs by down-regulating the CaMKII/STAT1 signaling pathway.

**Conclusions:**

TGF-β1 induces an LTCC-mediated Ca^2+^ response, followed by activation of CaMKII/STAT1 signaling pathway, which promotes the pro-fibrotic functions of OFs and participates in fibrogenesis during TED. Nimodipine exerts potent anti-fibrotic benefits in vitro by suppressing the CaMKII/STAT1 signaling pathway. Our work deepens our understanding of the fibrogenesis process during TED and provides potential therapeutic targets and alternative candidate for TED.

**Supplementary Information:**

The online version contains supplementary material available at 10.1186/s40662-024-00401-5.

## Background

Thyroid eye disease (TED), also known as Graves’ orbitopathy, is a complex organ-specific disorder [1–6]. The prevalence of TED is highest among patients with Graves’ disease (GD) and the overall pooled prevalence is 40% (CI 0.32 to 0.48) [7]. Clinically, patients with TED commonly experience exophthalmos and diplopia, significantly impacting their quality of life [2, 8]. In severe cases, irreversible visual impairment may occur due to exposure keratopathy or compressive optic nerve disorders in the context of dysthyroid optic neuropathy (DON) [9–11]. The pathological process of TED mainly comprises orbital inflammation and persistent fibrogenesis [12]. In the active phase, the disease is characterized by orbital inflammation, escalating oxidative stress, and activation of orbital fibroblasts (OFs), subsequently leading to increased adipogenesis, excessive production of hyaluronan, myofibroblast differentiation, and eventual tissue fibrosis [11, 13]. Orbital tissue fibrosis takes major responsibility for the intractable complications in the late stage of TED [8, 14]. The existing therapeutic approaches for TED mainly focus on alleviating orbital inflammation and oxidative stress in patients with TED; however, their efficacy in ameliorating orbital tissue fibrosis remains uncertain [2, 3, 15–21]. The clinical demand for effectively inhibiting fibrogenesis during TED is still unmet. Therefore, it is imperative to investigate the molecular mechanisms underlying TED orbital tissue fibrosis and identify potent anti-fibrotic agents.

So far, fibrogenesis during TED remains incompletely understood. Previous studies have suggested that OFs are crucial targets and effector cells in TED [22], and the myofibroblast transdifferentiation, proliferation and migration of OFs induced by transforming growth factor-beta 1 (TGF-β1) represent crucial processes in fibrogenesis during TED [8, 11, 23–25]. However, the TGF-β1 inductive pro-fibrotic mechanisms in TED have not been fully elucidated. Further studies of TGF-β1 signaling may help develop novel therapeutic strategies targeting fibrosis in TED. Calcium ions (Ca^2+^) are a versatile signaling intermediate essential for a wide range of cellular biological processes including contraction, secretion, metabolism, proliferation, and differentiation [26]. Recent work suggests that Ca^2+^ signaling is involved in the signal transduction of TGF-β1 and participates in fibrotic events occurring in several tissues including the heart, lung, liver, kidney and conjunctiva [27–31]. The latest study also uncovered that Ca^2+^ signaling may contribute to TED adipogenesis through its correlation with platelet-derived growth factor receptor [32]. Consequently, whether an alteration in Ca^2+^ signaling contributes to fibrogenesis during TED is worth exploring.

The L-type calcium channel (LTCC) is known to be critical in supplementing cytoplasmic Ca^2+^ as well as triggering downstream signaling pathways in excitable cells [33, 34]. Of interest, recent studies have revealed that the dominant subunit of LTCC is also expressed on some non-excitable cells and regulates vital activities [34–38]. Therefore, it is important to determine whether LTCC plays a role in the regulation of OFs function. Nimodipine, a highly selective inhibitor of LTCC, has been shown to distribute well in the ocular circulation with good tolerability [39–43]. Additionally, recent work suggests that nimodipine has immunoregulatory effects [44, 45], which is also validated by our preclinical study [46]. In this report, we investigate the potential role of Ca^2+^ signaling in fibrogenesis during TED and evaluate the therapeutic effects of nimodipine through a TGF-β1 induced in vitro TED model.

## Methods

### Participant enrollment and tissue collection

Orbital adipose connective tissues were consecutively obtained from 6 patients with TED who underwent decompression surgery at Zhongshan Ophthalmic Center and Sun Yat-sen Memorial Hospital. The diagnostic criteria for TED refer to those developed by Bartley and Gorman [47]. All patients with TED were inactive, kept euthyroid and discontinued glucocorticoid therapy for at least 3 months. Also, history of orbital irradiation was forbidden. Orbital adipose connective tissues were also collected as surgery wastes from 6 healthy control (HC) donors who underwent blepharoplasty (n = 4) or surgery for orbital trauma (n = 2) at Zhongshan Ophthalmic Center. All the HC donors were free of any thyroid disease and TED. All enrolled participants signed informed consent forms, and were devoid of other systemic autoimmune diseases, infectious diseases, other fibrotic disorders, and malignant diseases. Clinical characteristics of these participants were summarized in Table 1. This study was conducted according to the Helsinki Declaration and approved by the institutional ethics committees of Zhongshan Ophthalmic Center (2020KYPJ104) and Sun Yat-sen Memorial Hospital (2020-KY-122).

**Table 1:** Baseline characteristics of patients with TED and HC donors

Characteristics	Patients with TED	HC donors
Patients, n	6	6
Sex, male/female	3/3	3/3
Age, mean ± SD, years	50.83 ± 5.55	43.33 ± 13.61
History of smoking, n	3	1
Duration of illness, median (IQR), months	43.5 (39.75–51.25)	N/A
History of thyroid disease, n		
Graves’ disease	6	N/A
Autoimmune thyroiditis	0	N/A
Others	0	N/A
Previous thyroid treatments, n		
Drugs	6	N/A
Radioiodine	1	N/A
Thyroidectomy	1	N/A
Clinical Activity Score (CAS)^a^, n		
Inactive	6	N/A
Active	0	N/A
Disease severity^b^, n		
Mild	0	N/A
Moderate to severe	6	N/A
Sight-threatening	0	N/A

*TED =* thyroid eye disease; *HC =* healthy control; *SD =* standard deviation; *n =* number; *N/A =* not applicable

^a^CAS was graded according to the 7-item Clinical Activity Score (CAS), CAS ≥ 3 indicates active and CAS < 3 indicates inactive

^b^Disease severity was determined according to the European Group on Graves’ orbitopathy (EUGOGO) classification, which defines the severity as mild, moderate-to-severe, or sight-threatening

All the specimens obtained from patients with TED and HC donors were utilized for paraffin-embedded sections. Due to the limited size of each specimen, the remaining specimens from 4 patients with TED (2 male, 2 female) and 4 HC donors (2 male, 2 female) were utilized for primary culture of OFs and subsequent experiments.

### Primary cell culture and treatments

Primary culture of OFs were performed as previously described [48, 49]. Briefly, orbital tissues were cut into small pieces (less than 1 × 1 mm) after removal of blood vessels and placed in T25 flasks. A mixture of Dulbecco’s Modified Eagle Medium/Ham’s Nutrient Mixture F-12 (DMEM/F12; 1:1 ratio) supplemented with 20% fetal bovine serum (FBS) and 1% penicillin/streptomycin (all from Gibco Laboratories, New York, USA) was added, and flasks were incubated in a humidified incubator at 37 °C with 5% CO_2_. OFs were harvested when cells reached 80% confluence and then passaged using 0.25% trypsin/EDTA. Subsequently, OFs were cultured in DMEM/F12 supplemented with 10% FBS and antibiotics following standard cell culture protocols [48, 49]. OFs were used between the third and the eighth passages in following in vitro experiments. Nimodipine, KN-93 Phosphate (KN-93), fludarabine (all obtained from Selleck Chemicals, Houston, TX, USA) and TGF-β1 (PeproTech Inc., Rocky Hill, NJ, USA) were applied for cell treatments upon different conditions (relevant details are provided in the following methods section).

### Cytotoxicity assay

OFs were seeded in 96-well plates and treated with 20–100 μmol/L nimodipine, 5–40 μmol/L KN-93, or 5–40 μmol/L fludarabine separately. The cytotoxicity assay was performed using a cell counting kit-8 (CCK-8) (Beyotime Biotechnology, Shanghai, China) according to the manufacturer’s instructions. The results were expressed as percentages of untreated control values and presented as mean ± standard deviation (SD).

### RNA sequencing and gene expression omnibus (GEO) dataset analysis

OFs were co-cultured with TGF-β1 for 24 h with or without 60 μmol/L nimodipine pretreatment (5 min). OFs without nimodipine pretreatment and TGF-β1 served as the control group (n = 3, each group). The total RNA was extracted using a Direct-zol RNA MicroPrep Kit (Zymo Research, Irvine, CA, USA). The Yale Center for Genome Analysis used a Ribo-Zero rRNA Removal Kit (Illumina Co. San Diego, CA, USA) to process the total RNA, construct libraries and perform standard Illumina HiSeq2000 sequencing, obtaining > 40 million reads per sample.

To perform gene ontology (GO) analysis, the up- or down-regulated genes were assigned with biological functions according to the Database for Annotation, Visualization, and Integrated Discovery (DAVID), as previously described [46]. The functional variation of GO analysis is displayed as lollipop charts using R package “ggplot2”.

To analyze differentially expressed genes (DEGs), the Gene Expression Omnibus (GEO) database was queried, and the RNA sequencing dataset for TED, GSE58331, was selected. The cells subsets were categorized according to the annotation in original data. The transcripts were analyzed by R (version 4.0.3) and DEGs were identified with a fold change greater than 0.5 and *P* value less than 0.05. The expression of crucial genes that encode LTCC subunits is displayed as a heatmap using the R package “pheatmap” [50].

To identify the transcriptional factor (TF) that was involved in the Ca^2+^ signaling pathway, the DEGs between the “TGF-β1 only” and “TGF-β1 + nimodipine pretreatment” groups were screened by AnimalTFDB (version 3.0) database. The DEGs underlain the TF were identified and are displayed in Heatmap using the R package “pheatmap”.

### Flow cytometry (FCM)

For intracellular free Ca^2+^ level measurement, OFs were digested and resuspended in Hanks’ solution with 2 mM calcium, and then loaded with 4 μmol/L Indo-1/AM (Invitrogen, Life Technologies, Carlsbad, CA, USA) for 30 min before flowcytometric analysis on an Aurora system (Cytek Biosciences, Fremont, CA, USA). FCM was performed to detect changes in the kinetics of Indo-1, the emission of which shifted from about 475 nm without Ca^2+^ to about 400 nm with Ca^2+^, and the data were presented as Indo-1 ratios [51]. The OFs derived from patients with TED (TED-OFs) were divided into three groups: (1) Vehicle group (applied with 1 μL trehalose solution, a solvent of TGF-β1, served as negative control), (2) TGF-β1 group (applied with 10 ng/mL TGF-β1), (3) Nimodipine pretreatment group (OFs were pretreated with 60 μmol/L nimodipine for 5 min, with no subsequent wash, before applying with 10 ng/mL TGF-β1). After 60 ± 10 s baseline recording, different stimulus (vehicle solution or TGF-β1 10 ng/mL) was added to FCM samples and analysis on the Aurora system for above 250 s.

For FCM of phospho-signal transducer and activator of transcription 1 [p-STAT1(Ser727)], TED-OFs and OFs of HC donors (HC-OFs) were both digested and washed, fixed with 4% paraformaldehyde and permeabilized with methanol, followed by staining with phycoerythrin (PE) conjugated antibodies against p-STAT1(Ser727) (Biolegend, San Diego, CA, USA) and analyzed on a LSR Fortessa (BD Biosciences, New York, NJ, USA).

FCM data were processed using software FlowJo (version 10.4, FlowJo Co., OR, USA).

### RNA isolation and real-time quantitative polymerase chain reaction (RT-qPCR)

Total RNA was isolated from OFs using an RNA-Quick Purification Kit (Yishan Biotechnology Co., Ltd, Shanghai, China). The total RNA was reverse-transcribed into cDNA using a HiScript II Q RT SuperMix for RT-qPCR (Vazyme, Nanjing, China). RT-qPCR was performed on a Roche Light-Cycler 480 system (Roche, Basel, Switzerland) using a ChamQ SYBR Color qPCR Master Mix (Vazyme). The relative expression levels of CACNA1C, CACNB2, CACNA2D1, α-SMA, Col1A1 and Col1A2 mRNA were analyzed by the 2^–ΔΔCt^ method. Glyceraldehyde-3-phosphate dehydrogenase (GAPDH) was used as the internal control. The gene-specific primer sequences for RT-qPCR (all obtained from Sangon Biotech, Shanghai, China) are listed in Table 2.

**Table 2:** Primer sequences of real-time quantitative polymerase chain reaction (RT-qPCR)

Genes	Sequences (5ʹ-3ʹ)
CACNA1C	F: TCCTCCGCTCTGCCTCACTAG
	R: CACTGCCAATGCCTGATGATGAAC
CACNA2D1	F: ACTGCTGCTGCCTGGTCTATTC
	R: ATCCTCCATCTCAACTGCCTCAAG
CACNB2	F: CGCTCCTATCCGTTCTGCTTCC
	R: TCCTGGGTTTCCGAGTCAAATGTC
α-SMA	F: CTCTGGACGCACAACTGGCAC
	R: CACGCTCAGCAGTAGTAACGAAGG
Col1A1	F: AAAGATGGACTCAACGGTCTC
	R: CATCGTGAGCCTTCTCTTGAG
Col1A2	F: CTCCATGGTGAGTTTGGTCTC
	R: CTTCCAATAGGACCAGTAGGAC
GAPDH	F: TTGCCATCAATGACCCCTT
	R: CGCCCCACTTGATTTTGGA

### 5-ethynyl-2′-deoxyuridine (EdU) proliferation assay

OFs were seeded in 12-well plates at a density of 5 × 10^4^ per well overnight, and then pretreated with nimodipine (20, 40, or 60 μmol/L) or KN-93 (10 μmol/L), with no subsequent wash, followed by 10 ng/mL TGF-β1 stimulation for 24 h. After treatments, the cells were labeled with a click reaction cocktail using an EdU assay kit (Beyotime Biotechnology, Shanghai, China) according to the manufacturer’s instructions. The images were captured using an inverted fluorescent microscope (Nikon, Tokyo, Japan). Percentages of EdU-positive OFs were counted. The results were averaged in each group (n = 4), and finally expressed as the ratio of “EdU + cells” to “Total number cells”. FCM analysis of EdU + OFs was also performed with a LSR Fortessa (BD Biosciences) and the data were processed using software FlowJo (version 10.4).

### Wound healing assay

OFs were seeded in 6-well plates at a density of 1 × 10^5^ per well overnight. The OFs were then pretreated with nimodipine at concentrations of 20, 40, or 60 μmol/L, or KN-93 at a concentration of 10 μmol/L only, with no subsequent wash. Confluent cell monolayers were wounded by a pipette tip and a straight scratch was made. Wound width was assessed at 0, 12 and 24 h. Wound closure images were captured with a microscope camera (Canon, Tokyo, Japan). The results were expressed as the wound width.

### Western blot (WB) analysis

Proteins of OFs with different treatments and orbital adipose connective tissues from both patients with TED and HC donors were extracted in RIPA lysis buffer (KeyGEN Biotech, Jiangsu, China) and the concentrations were quantified using a BCA assay reagent kit (Beyotime) according to manufacturer’s instructions. WB was conducted as previously described [52]. After blocking, the polyvinylidene difluoride (PVDF) membranes were incubated with primary antibodies against Col1A1, phospho-CaMKII (p-CaMKII), β-tubulin, GAPDH (all obtained from Cell Signaling Technology, Boston, MA, USA), α-SMA, CaMKII, p-STAT1 (ser727), STAT1 (all obtained from Abcam, Cambridge, UK), and Flag (ProteinTech), followed by incubation with appropriate secondary antibodies (Cell Signaling Technology). WB were imaged and grayscale values were quantified by ImageJ (NIH, Bethesda, MD, USA), and normalized to GAPDH expression levels.

### Immunohistochemistry (IHC)

Paraffin sections (4 μm) of orbit adipose connective tissues were made. All the specimens obtained from patients with TED and HC donors were utilized for IHC assay of p-CaMKII (Thr286/287) and CaMKII. Due to the limited size of each specimen, remaining paraffin sections from 5 patients with TED (2 male, 3 female) and 5 HC donors (2 male, 3 female) were utilized for probing the presence and expression of p-STAT1 (ser727) and STAT1 using IHC. After dewaxing, rehydration and antigen retrieval, the sections were incubated with p-CaMKII (Thr286/287), CaMKII, p-STAT1 (ser727) or STAT1 antibodies (all Abcam) overnight at 4 °C, followed by the appropriate secondary antibodies and diaminobenzidine (DAB) at room temperature. Photographs were captured using a microscope camera (Carl-ZEISS, Oberkochen, Germany). Images were subjected to IHC scoring as previously described [53] by two clinicians (QC and YWH) according to the following criteria: 0, no staining, 1, faint, cytoplasmic and nuclear staining, 2, moderate, smooth cytoplasmic and nuclear staining, 3, intense, granular cytoplasmic and nuclear staining.

### Co-immunoprecipitation (Co-IP) assay

The plasmids of pcDNA 3.1-STAT1-FLAG and the corresponding empty vector were purchased from TranSheep Bio Co. Ltd (Shanghai, China) then verified by sequencing. OFs were transiently transfected with the plasmids using Lipofectamine 2000 (Invitrogen, Life Technologies, Carlsbad, CA, USA) according to the manufacturer’s instructions. After 48 h, cells were lysed, and the supernatant was collected after centrifugation. 10% of supernatant was preserved as the whole cell lysate (WCL), and the rest was subjected to Co-IP. Co-IP was conducted with mouse anti-Flag monoclonal antibody (PeproTech Inc., Rocky Hill, NJ, USA) on a rotary table overnight at 4 °C, followed by incubation with protein A agarose beads (Cell Signaling Technology, Boston, MA, USA) for 4 h. After 3 washes, the precipitate was collected by centrifugation and resuspended in RIPA lysis buffer. Denatured proteins were separated as previously described [52]. Primary rabbit antibodies against Flag (ProteinTech), CaMKII (Abcam) and GAPDH (Cell Signaling Technology) were incubated with the PVDF membrane, followed by incubation with the appropriate secondary antibody (Cell Signaling Technology). WB bands were imaged as previously described [52].

### Statistical analysis

All experiments were performed consecutively from at least 3 and up to 6 different individuals. Data from independent experiments were displayed as mean ± SD. Statistical analyses of one-way ANOVA, two-way ANOVA, or Student’s t-tests were conducted using GraphPad Prism (version 9, GraphPad Software, Inc. San Diego, CA, USA) where appropriate. A *P* value of less than 0.05 was considered statistically significant.

## Results

### Pivotal genes encoding LTCC are expressed in OFs

Based on previous Ca^2+^ signaling studies [34–38], we conducted RNA sequencing to investigate the expression of genes encoding crucial LTCC subunits [54] in TED-OFs, including Ca_v_α1 (corresponding genes were CACNA1S, CACNA1C, CACNA1D, and CACNA1F), Ca_v_β2 (corresponding gene: CACNB2) and Ca_v_α2δ (corresponding genes: CACNA2D1-4). The results revealed a high abundance of gene expression of Ca_v_1.2α1 (CACNA1C) and Ca_v_α2δ-1 (CACNA2D1) in TED-OFs. A low abundance of gene expression of Ca_v_β2 (CACNB2) was also observed, while the expression levels of Ca_v_1.3α1 (CACNA1D) and the remaining genes encoding Ca_v_α2δ subunits (CACNA2D2-4) were extremely low. No gene expression was detected for Ca_v_1.1α1 and Ca_v_1.4α1 (corresponding to CACNA1S and CACNA1F, respectively) (Additional file 1: Table S1). These results provided evidence that essential genes that encode LTCC were expressed on TED-OFs.

**Fig. 1:**
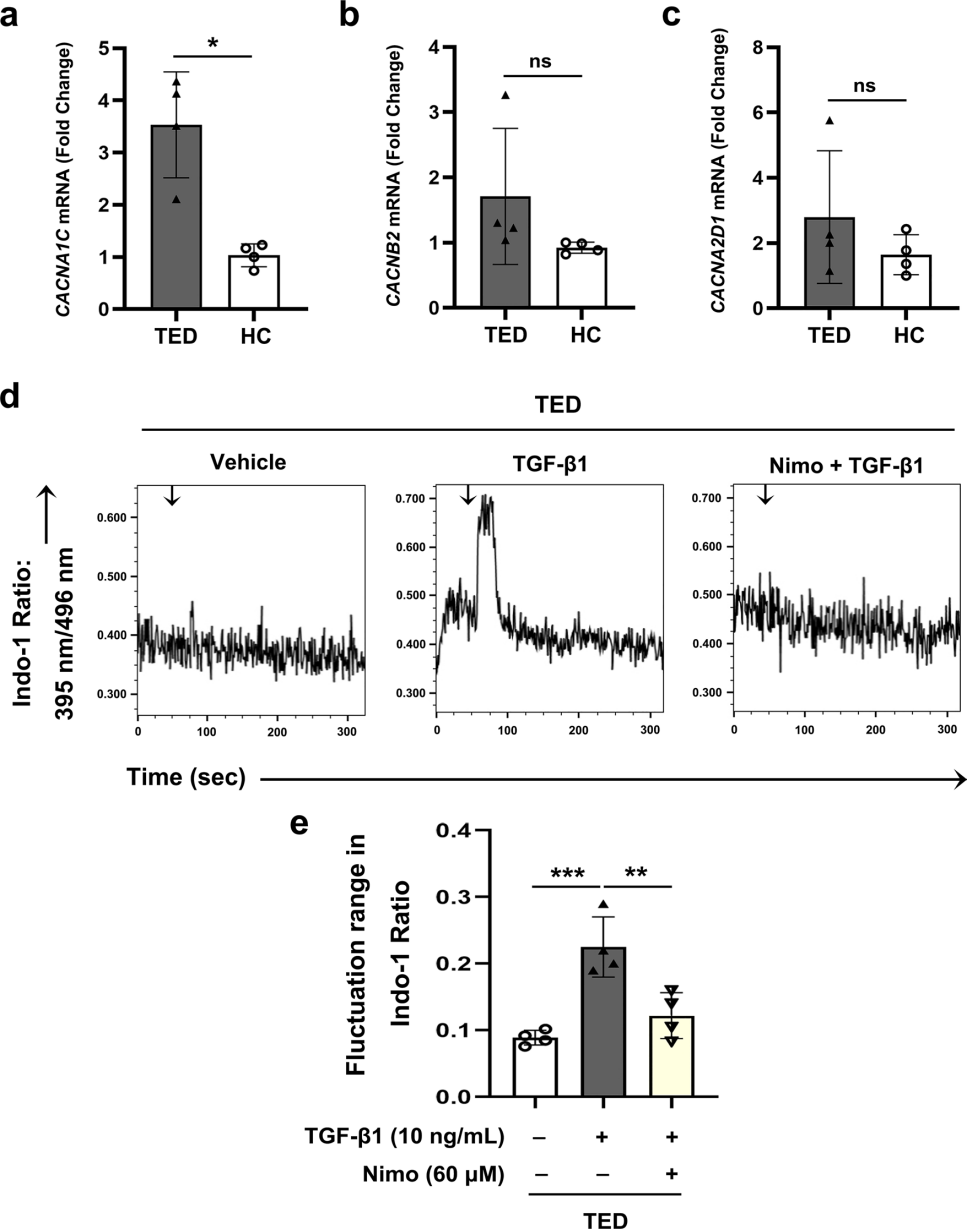
Pivotal genes encoding LTCC are expressed in OFs. LTCC mediates the intracellular Ca^2+^ response induced by TGF-β1. **a**–**c** The mRNA expression of CACNA1C, CACNB2 and CACNA2D1 in TED-OFs and HC-OFs in passage 3 were evaluated by RT-qPCR, n = 4. **d**–**e** The representative data and statistical analysis of the Indo-1 ratio in different groups detected by flow cytometry. After 60 ± 10 s baseline recording, different stimulus (trehalose solution, or TGF-β1 10 ng/mL) was added to flow cytometry samples (as indicated by the arrows). TGF-β1 induced a substantial elevation in the Indo-1 ratio and nimodipine (60 μmol/L) inhibited the TGF-β1 induced elevation in the Indo-1 ratio (n = 4, each group). The significance was determined using unpaired Student’s t-test (**a**–**c**), or one-way ANOVA (**e**). LTCC, L-type calcium channel; OF, orbital fibroblast; TGF-β1, transforming growth factor-beta 1; TED, thyroid eye disease; TED-OFs, OFs derived from patients with TED; HC, healthy control; HC-OFs, OFs derived from HC donors; RT-qPCR, real-time quantitative polymerase chain reaction; Nimo, nimodipine; *P < 0.05; **P < 0.01; ***P < 0.001; ns, not significant

We next conducted RT-qPCR to compare the expression levels of CACNA1C, CACNA2D1 and CACNB2 between TED-OFs and HC-OFs. In TED-OFs, there was an upregulation of CACNA1C, a core gene responsible for the majority of functions of LTCC, when compared with HC-OFs (Fig. 1a). However, the other two genes (CACNB2 and CACNA2D1) associated with auxiliary subunits of LTCC showed no significant difference in expression between the two groups (Fig. 1b, c). Additionally, RNA sequencing data from GSE58331 support our findings by illustrating that CACNA1C was up-regulated in patients with TED, yet other genes encoding LTCC subunits showed no significant difference between the two groups (Additional file 2: Fig. S1).

Collectively, our findings suggest that pivotal genes encoding LTCC are expressed in OFs, and the up-regulated expression of CACNA1C may play a role in the pathogenesis of TED.

### ***LTCC mediates TGF-β1 induced intracellular Ca***^***2***+^***response***

To explore whether Ca^2+^ response participates in the pro-fibrotic effects of TGF-β1 in TED, Indo-1/AM (Indo-1) [51] was used to assess the changes of intracellular Ca^2+^ levels after TGF-β1 stimulation. The results showed that TGF-β1 induced a significant quick elevation of the Indo-1 ratio in TED-OFs (Fig. 1d, e), indicating a quick increase of intracellular free Ca^2+^ on TGF-β1 stimulation. Moreover, when pretreated with nimodipine, a highly selective inhibitor of LTCC, there was no noticeable elevation in the Indo-1 ratio following TGF-β1 stimulation in TED-OFs (Fig. 1d, e), suggesting that LTCC contributed to intracellular free Ca^2+^ increase induced by TGF-β1. The CCK-8 assay showed that concentrations of nimodipine below 100 μmol/L were safe for OFs (Additional file 3: Fig. S2).

Taken together, these results provide evidence that LTCC mediates TGF-β1 induced intracellular Ca^2+^ response.

### Nimodipine attenuates pro-fibrotic gene expression levels in OFs

**Fig. 2:**
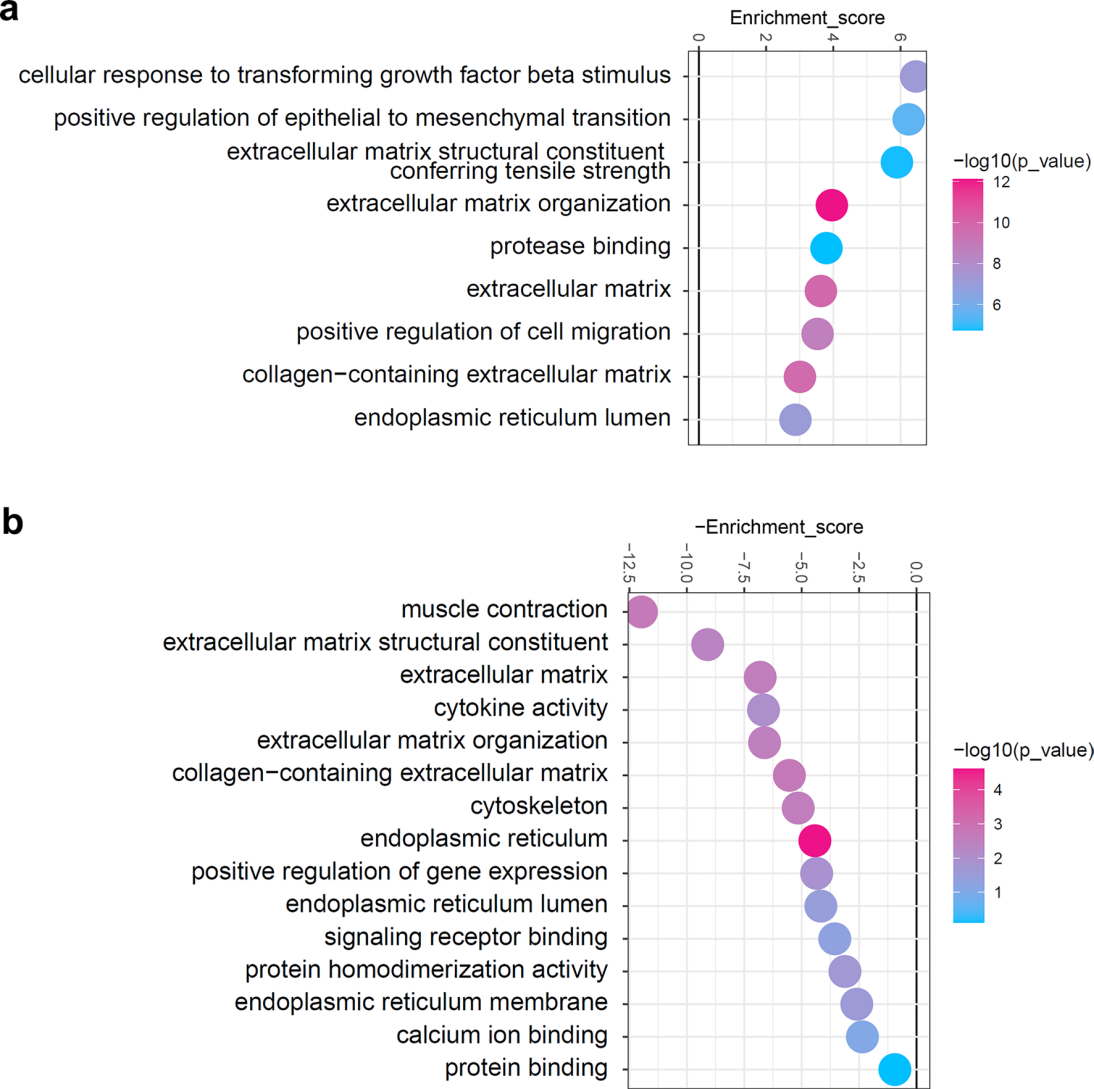
Bioinformatics analysis results. **a**, **b** Lollipop plot showing the representative gene ontology terms enriched in fibrosis-associated functions based on functional enrichment analysis. **a** Up-regulated functionsin the transforming growth factor-beta 1 (TGF-β1) group (n = 3); **b** Down-regulated functions in the nimodipine pretreatment group (n = 3)

Since we found that the selective LTCC inhibitor nimodipine effectively reduced the intracellular Ca^2+^ response induced by TGF-β1 in TED-OFs, further studies were conducted to assess the potential anti-fibrotic effects of nimodipine in vitro. Firstly, we performed RNA sequencing to compare DEGs of TED-OFs co-culture with TGF-β1 with or without nimodipine pretreatment. Previous studies suggested that excessive synthesis of collagen I and expression of α-SMA are the key features of the myofibroblast transdifferentiation of OFs [8, 21], and enhanced cell migration further facilitate the progression of fibrosis in TED [55–57]. Consistent with previous work, bioinformatics results showed that TGF-β1 elicited a pathogenic fibrotic phenotype in TED-OFs, characterized by an up-regulation in collagen production and formation, and enhanced cell migration (Fig. 2a). When pretreated with nimodipine, the collagen-containing extracellular matrix (ECM) synthesis induced by TGF-β1 was significantly attenuated, and the structural constituent and organization of ECM were both notably decreased (Fig. 2b). A reduction in Ca^2+^ ion binding and diminished signaling transduction were also observed in the nimodipine pretreatment group.

**Fig. 3:**
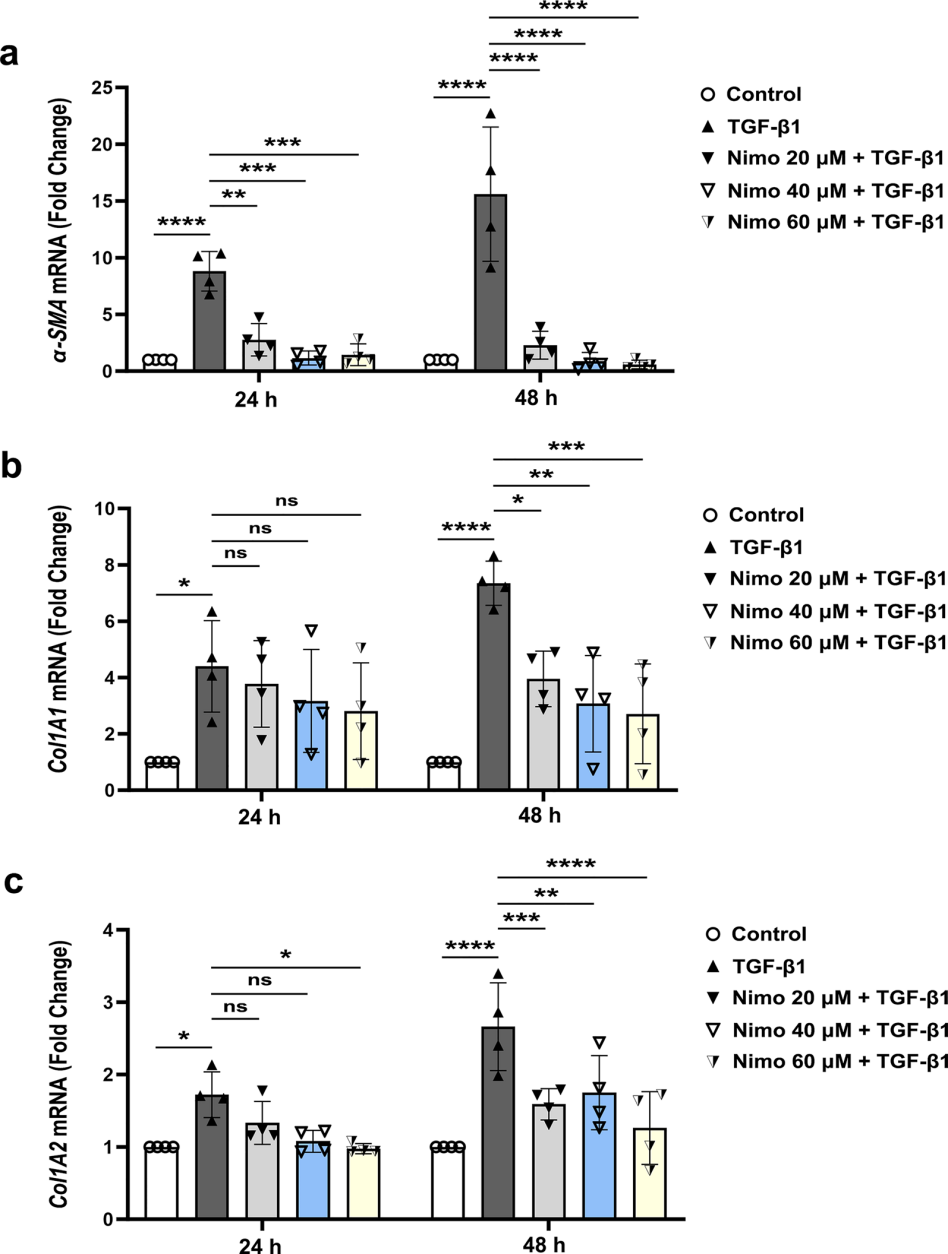
Nimodipine attenuates the expression of pro-fibrotic genes induced by TGF-β1 in OFs. **a**–**c** TED-OFs were pretreated with 0 (control), 20, 40 or 60 μmol/L nimodipine for 5 min with no subsequent wash, followed by 10 ng/mL TGF-β1 stimulation for 24 or 48 h. The mRNA expression of α-SMA, Col1A1, and Col1A2 were evaluated by RT-qPCR. Results were normalized with GAPDH levels, n = 4, one-way ANOVA. TGF-β1, transforming growth factor-beta 1; TED-OFs, OFs derived from patients with thyroid eye disease; α-SMA, alpha-smooth muscle actin; Col1A1, collagen type I alpha 1; Col1A2, collagen type I alpha 2; RT-qPCR, real-time quantitative polymerase chain reaction; GAPDH, glyceraldehyde-3-phosphate dehydrogenase; Nimo nimodipine; *P < 0.05; **P < 0.01; ***P < 0.001; ****P < 0.0001; ns, not significant

To further confirm the anti-fibrotic effects of nimodipine, we compared the gene expression of α-SMA and collagen I in cultured TED-OFs under different specified conditions. RT-qPCR results showed that TGF-β1 increased the pro-fibrotic gene expression levels of α-SMA, Col1A1 and Col1A2 in TED-OFs. Moreover, all the abovementioned pro-fibrotic genes were down-regulated in a dose-dependent manner upon pretreatment with nimodipine (Fig. 3a–c).

These findings collectively suggest that nimodipine effectively attenuates the expression of pro-fibrotic genes induced by TGF-β1 in TED-OFs.

### Nimodipine inhibits cell proliferation and migration of OFs

**Fig. 4:**
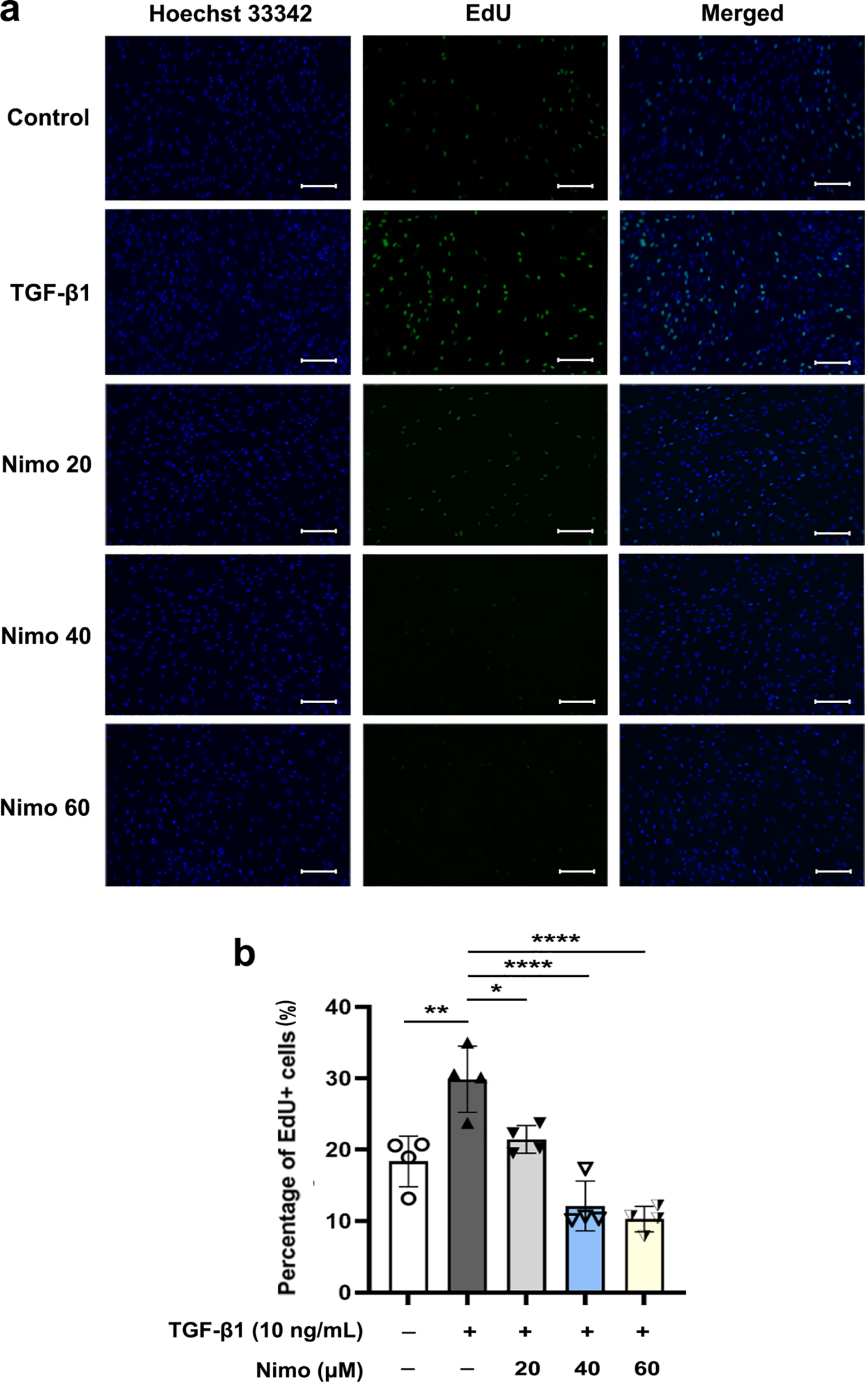
Nimodipine alleviated TGF-β1 induced cell proliferation of OFs. **a**, **b** The representative images and statistical analysis of EdU-positive TED-OFs in different groups are shown. Before EdU assay, OFs were pretreated with 0 (control), 20, 40 or 60 μmol/L nimodipine for 5 min, followed by 10 ng/mL TGF-β1 stimulation for 24 h. One-way ANOVA, n = 4; *P < 0.05; **P < 0.01; ****P < 0.0001. TGF-β1, transforming growth factor-beta 1; OF, orbital fibroblast; TED-OFs, OFs derived from patients with thyroid eye disease; EdU, 5-ethynyl-2′-deoxyuridine; Nimo, nimodipine. Scale bar, 100 μm

**Fig. 5:**
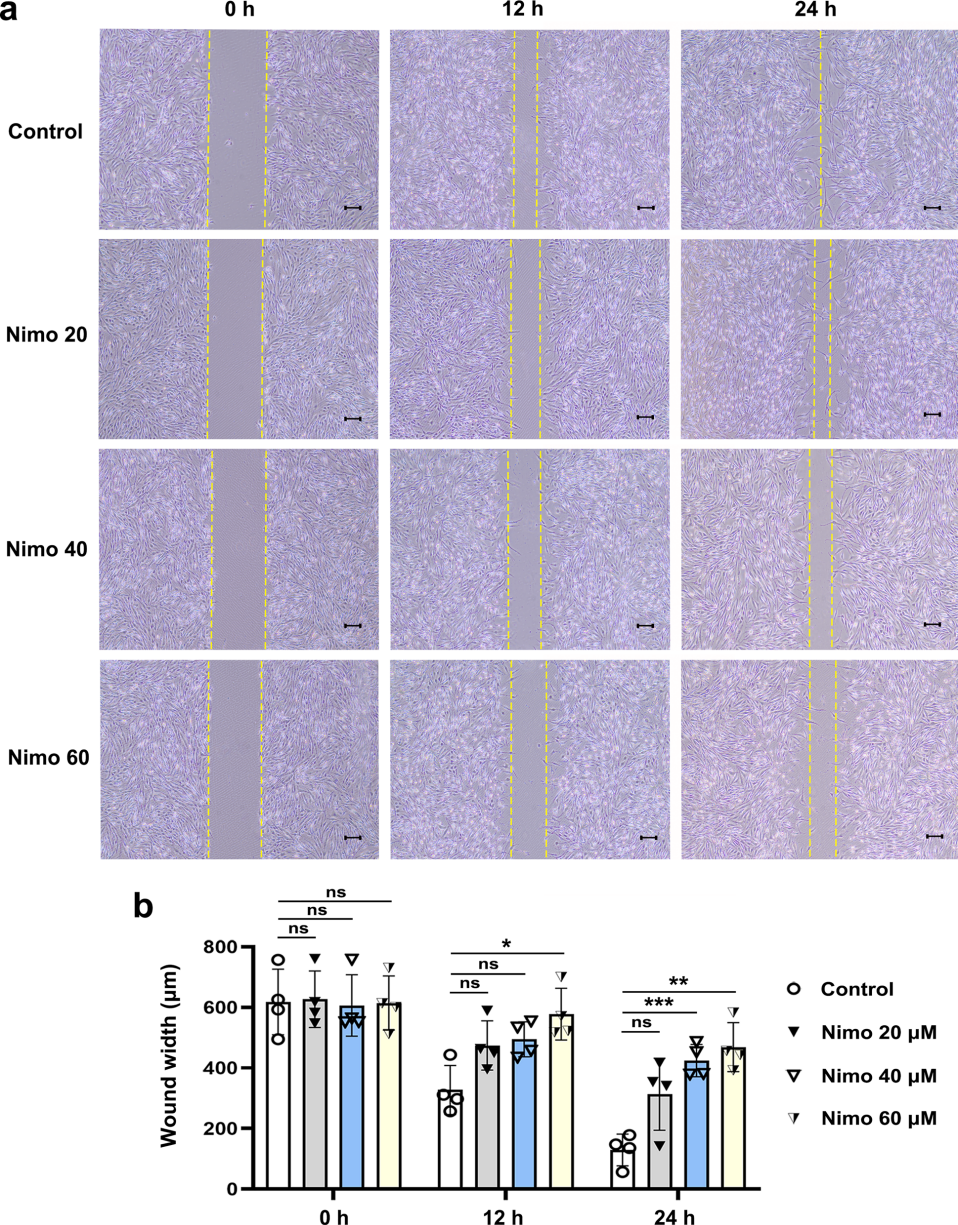
Nimodipine suppresses cell migration of OFs. **a**, **b** The representative wound healing images and statistical analysis of wound width in different groups of TED-OFs are shown. Before the wound healing assay, OFs were pretreated with 0 (control), 20, 40 or 60 μmol/L nimodipine for 5 min. One-way ANOVA, n = 4; *P < 0.05; **P < 0.01; ***P < 0.001; ns, not significant. OF, orbital fibroblast; TED-OFs, OFs derived from patients with thyroid eye disease; Nimo, nimodipine. Scale bar, 200 μm

Enhanced proliferation and migration of OFs have been shown to play a crucial role in fibrogenesis during TED [11, 23, 55–58]. Subsequently, the effects of nimodipine on the proliferation and migration of TED-OFs were assessed. The EdU assay revealed that TGF-β1 significantly induced cell proliferation as evidenced by a notable increase in the number of EdU-positive OFs. When pretreated with nimodipine, the TGF-β1 induced cell proliferation was alleviated dose-dependently, suggesting that LTCC plays an important role in mediating cellular proliferation of TED-OFs (Fig. 4a, b and Additional file 4: Fig. S3). Considering the pro-proliferative effect of TGF-β1, OFs were not co-cultured with TGF-β1 during the wound healing assay. We noticed delayed wound closure with pretreatment of 40 μmol/L and 60 μmol/L nimodipine, suggesting that LTCC mediated cellular migration of TED-OFs (Fig. 5a, b).

Collectively, these findings implicate LTCC in mediating cellular proliferation and migration of TED-OFs, and nimodipine alleviates the enhanced cell proliferation induced by TGF-β1 and inhibited the cell migration of TED-OFs.

### Aberrant CaMKII activation is involved in fibrogenesis during TED

**Fig. 6:**
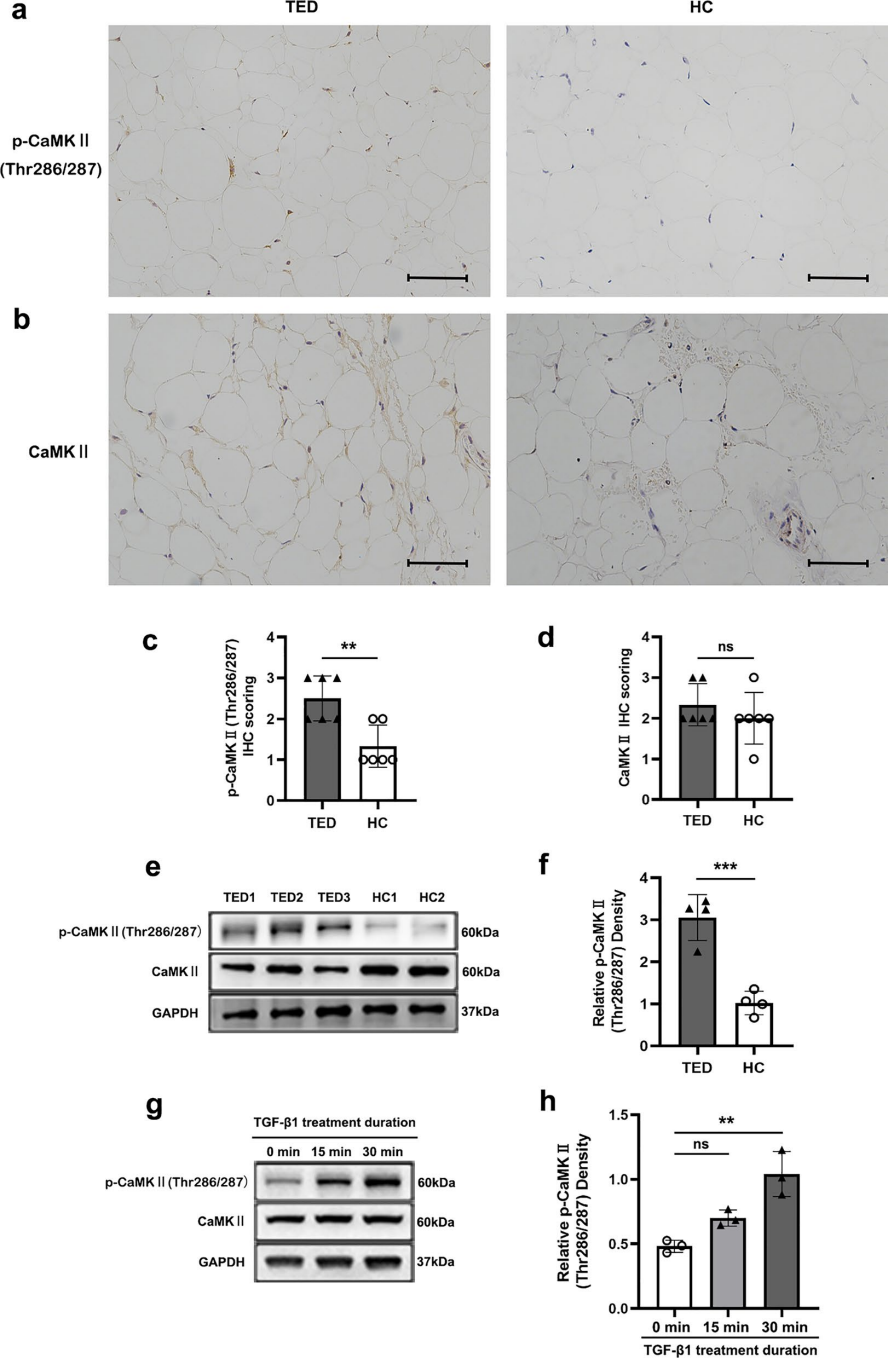
An aberrant CaMKII activation is involved in fibrogenesis during TED. **a**, **b** Representative IHC staining for p-CaMKII (Thr286/287) (**a**) and total CaMKII (**b**) were performed on paraffin-embedded biopsy sections obtained from orbital adipose connective tissues of patients with TED and HC donors. Scale bar, 100 μm. **c**, **d** Analysis of IHC scoring for p-CaMKII (Thr286/287) (**c**) and total CaMKII (**d**), n = 6. **e**, **f** WB showed up-regulated expression of p-CaMKII (Thr286/287) in the orbital adipose connective tissues of patients with TED compared to HC donors, n = 4. **g**, **h** WB showed up-regulated expression of p-CaMKII (Thr286/287) in response to TGF-β1 stimulation in TED-OFs, n = 3. The significance was determined by unpaired Student’s t-test (**c**, **d**, **f**) or one-way ANOVA (**h**). **P < 0.01; ***P < 0.001; ns, not significant. CaMKII, Ca^2+^/calmodulin-dependent protein kinase II; TED, thyroid eye disease; HC, healthy control; IHC, immunohistochemistry; WB, western blot analysis; TGF-β1, transforming growth factor-beta 1; TED-OFs, OFs derived from patients with TED; GAPDH, glyceraldehyde-3-phosphate dehydrogenase

Ca^2+^/calmodulin-dependent protein kinase II (CaMKII) serves as a key modulator in transducing Ca^2+^ signals [59, 60]. Phosphorylation at Thr286/287 is an active form of CaMKII [59]. Since our results have identified that TGF-β1 triggered a Ca^2+^ response in TED-OFs, we wondered whether CaMKII activation played a role in fibrogenesis during TED. As shown in Fig. 6a–d, IHC revealed an increase in CaMKII phosphorylation in TED orbital adipose connective tissues. While the difference of total CaMKII between the two groups was not statistically significant; this finding was further confirmed by WB (Fig. 6e, f). In TED-OFs, TGF-β1 stimulation also induced an up-regulated phosphorylation of CaMKII (Fig. 6g, h). All these results support that aberrant CaMKII activation participates in fibrogenesis during TED.

### Targeting CaMKII signaling exerts anti-fibrotic effects

**Fig. 7:**
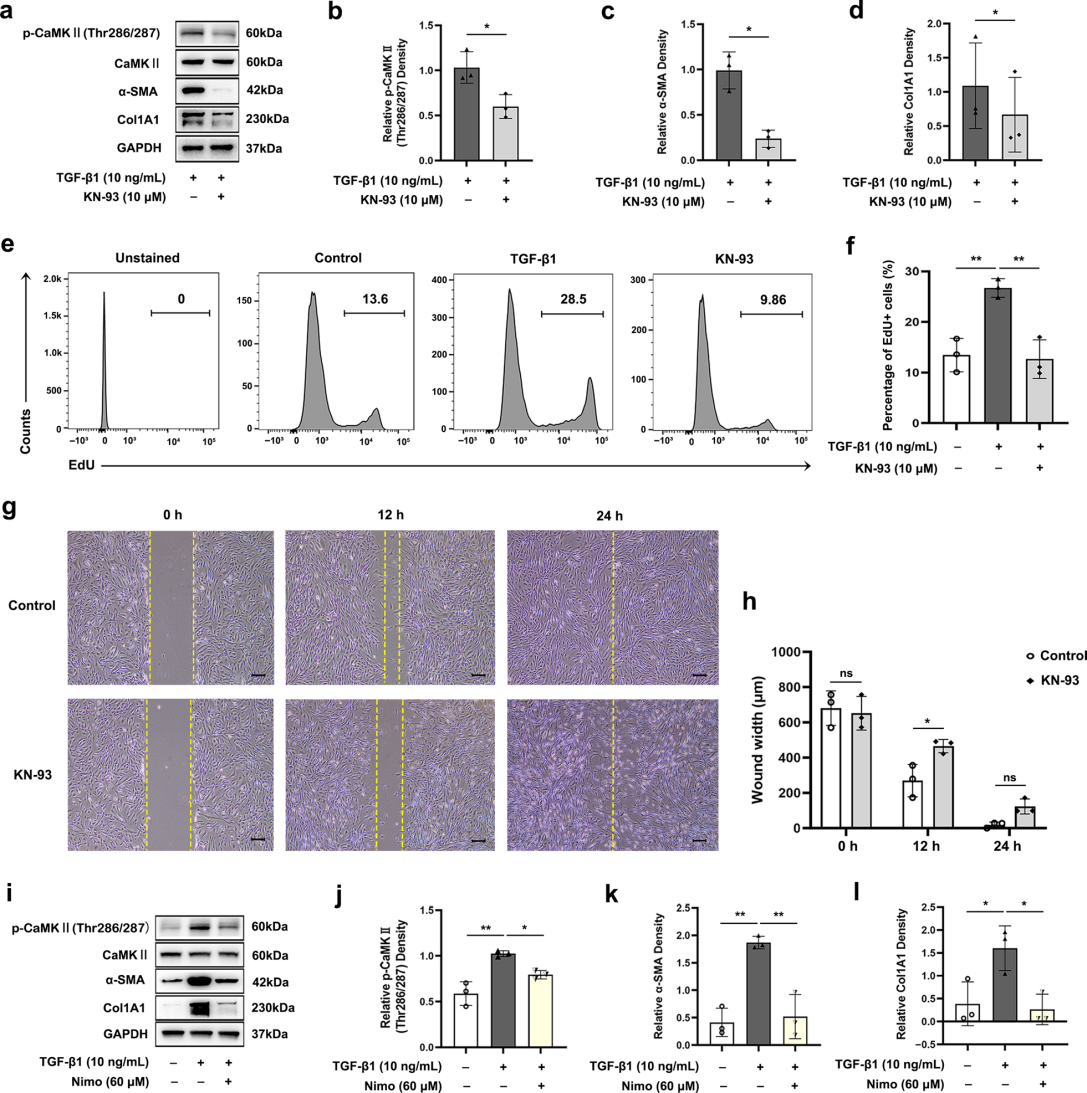
Targeting CaMKII signaling exerts anti-fibrotic effects. **a**–**d** WB showed that KN-93 phosphate pretreatment (10 μmol/L) for 1 h inhibited TGF-β1 induced p-CaMKII (Thr286/287) protein expression at 30 min and inhibited TGF-β1 induced α-SMA and Col1A1 protein expression at 48 h in TED-OFs, n = 3. **e**, **f** The representative data and statistical analysis of EdU-positive TED-OFs in different groups assessed by flow cytometry, n = 3. Before EdU assay, OFs were pretreated with 0 (control) or 10 μmol/L KN-93 phosphate for 1 h followed by 10 ng/mL TGF-β1 stimulation for 24 h. **g**, **h** The representative wound healing images and statistical analyses of wound width showed that KN-93 phosphate pretreatment (10 μmol/L) for 1 h delayed wound closure at 12 h in TED-OFs, n = 3. Scale bar, 200 μm. **i**–**l** WB showed that nimodipine pretreatment (60 μmol/L) suppressed TGF-β1 induced p-CaMKII (Thr286/287) protein expression at 30 min and inhibited TGF-β1 induced α-SMA and Col1A1 protein expression at 48 h in TED-OFs, n = 3. The significance was determined by unpaired Student’s t-test (**b**–**d**, **h**) or one-way ANOVA (**f**, **j**–**l**). *P < 0.05; **P < 0.01; ns, not significant. CaMKII, Ca^2+^/calmodulin-dependent protein kinase II; WB, western blot analysis; TGF-β1, transforming growth factor-beta 1; α-SMA, alpha-smooth muscle actin; Col1A1, collagen type I alpha 1; TED-OFs, OFs derived from patients with thyroid eye disease; OF, orbital fibroblast; EdU, 5-ethynyl-2′-deoxyuridine; GAPDH, glyceraldehyde-3-phosphate dehydrogenase; Nimo, nimodipine

Based on the above, subsequent investigations were undertaken to clarify the involvement of CaMKII signaling in fibrogenesis during TED. As shown in Fig. 7a–d, a specific CaMKII inhibitor, KN-93, attenuated the TGF-β1 induced phosphorylation of CaMKII as well as the protein expression levels of α-SMA and Col1A1 in TED-OFs. Furthermore, KN-93 suppressed TGF-β1 induced cell proliferation (Fig. 7e, f) and inhibited cell migration (Fig. 7g, h) of TED-OFs. CCK-8 assays showed that concentrations of KN-93 below 20 μmol/L were safe for OFs (Additional file 5: Fig. S4). When pretreated with nimodipine, the TGF-β1 induced phosphorylation of CaMKII was inhibited and the protein expression of α-SMA and Col1A1 were reduced (Fig. 7i–l).

Collectively, these results suggest an essential role of CaMKII signaling in TGF-β1 induced myofibroblast transdifferentiation and proliferation of TED-OFs, and CaMKII signaling is also involved in cellular motility of TED-OFs. Nimodipine exerts anti-fibrotic effects by down-regulating CaMKII signaling.

### Nimodipine suppresses the CaMKII/STAT1 signaling pathway to exert anti-fibrotic effects

**Fig. 8:**
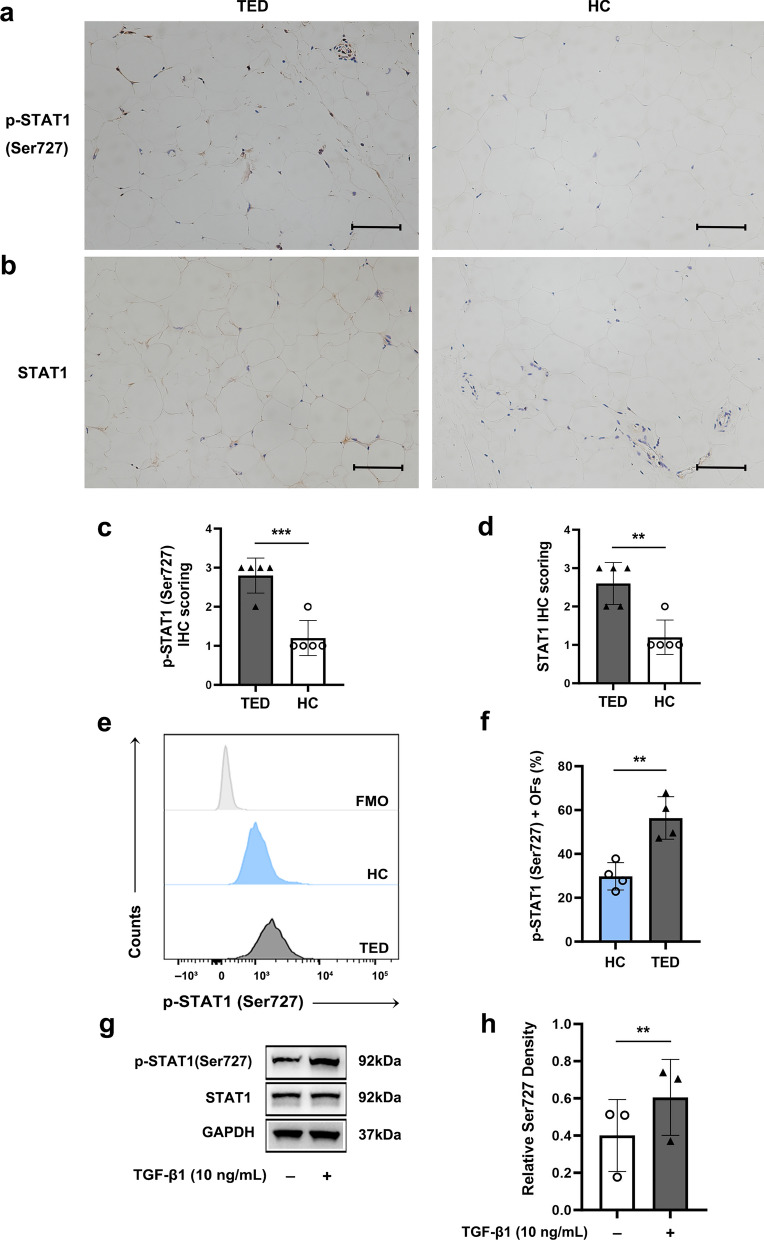
An activation of STAT1 is involved in fibrogenesis during TED. **a**, **b** Representative IHC staining for p-STAT1 (Ser727) (**a**) and total STAT1 (**b**) were performed on paraffin-embedded biopsy sections. Scale bar, 100 μm. **c**, **d** Analysis of IHC scoring of p-STAT1 (Ser727) (**c**) and total STAT1 (**d**), n = 5. **e**, **f** The representative data and statistical analysis of p-STAT1 (Ser727) levels of TED-OFs and HC-OFs detected by flow cytometry, n = 4. **g**, **h** WB confirmed up-regulated expression of p-STAT1(Ser727) in response to TGF-β1 stimulation in TED-OFs, n = 3. **P < 0.01, ***P < 0.001, unpaired Student’s t-test. STAT1, signal transducer and activator of transcription 1; TED, thyroid eye disease; HC, healthy control; IHC, immunohistochemistry, TED-OFs, OFs derived from patients with TED; HC-OFs, OFs derived from HC donors; WB, western blot analysis; TGF-β1, transforming growth factor-beta 1; FMO, fluorescence minus one; GAPDH, glyceraldehyde-3-phosphate dehydrogenase

Recent studies show that STAT1 signaling participates in tissue fibrosis [61–64]; CaMKII was also shown to modulate the activation of STAT1 [65, 66]. Interestingly, IHC identified an up-regulation in both the phosphorylation level and total expression of STAT1 in TED orbital adipose connective tissues, when compared to the HC group (Fig. 8a–d). FCM analysis further supported the IHC results by illustrating an elevated level of STAT1 phosphorylation in TED-OFs (Fig. 8e, f). Additionally, WB analysis revealed that TGF-β1 induced an up-regulation of STAT1 phosphorylation in TED-OFs (Fig. 8g, h). Taken together, these results demonstrate that the activation of STAT1 signaling is involved in fibrogenesis during TED.

**Fig. 9:**
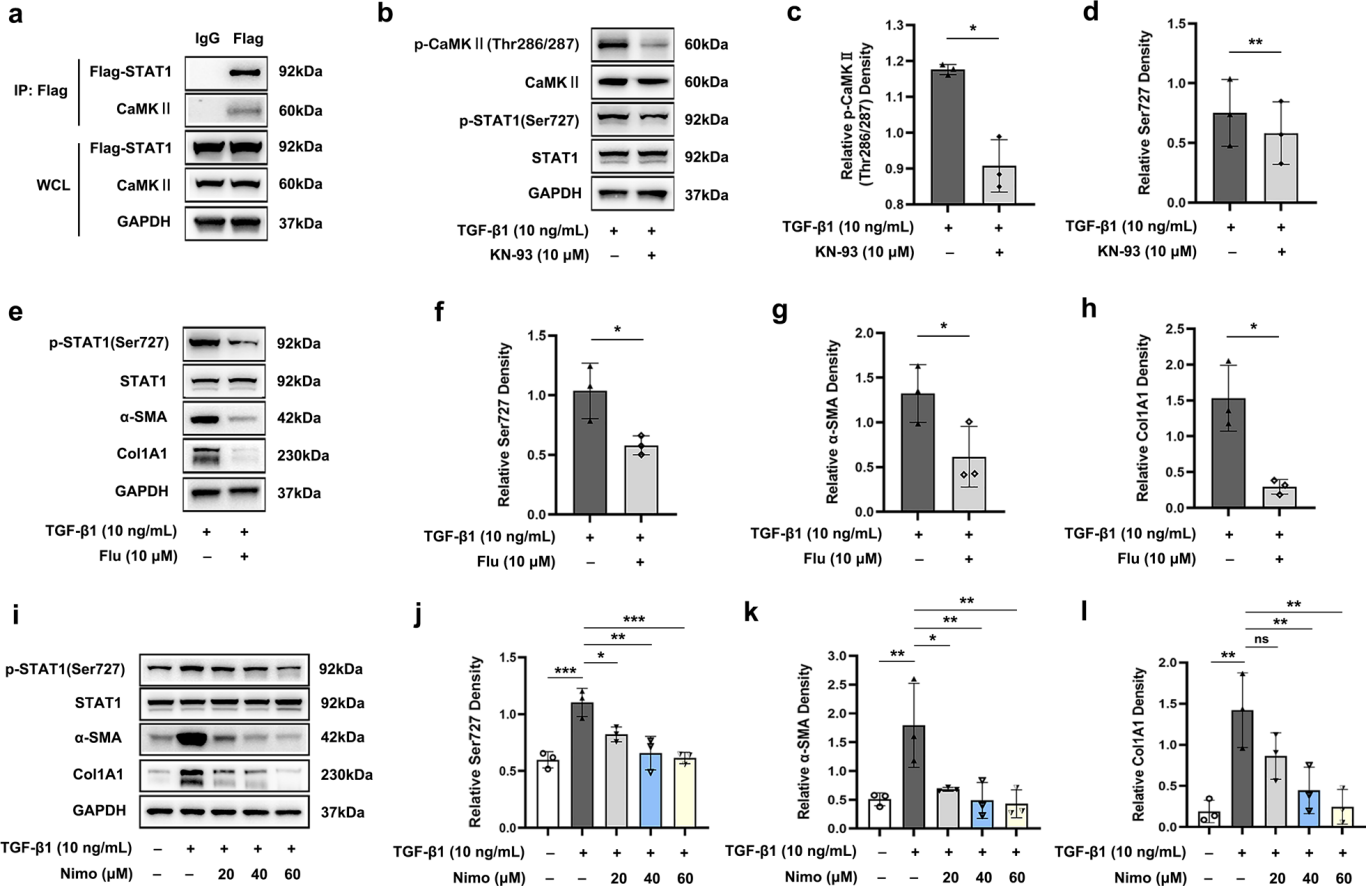
Nimodipine exerts anti-fibrotic effects by suppressing CaMKII/STAT1 signaling pathway in OFs. **a** Co-IP identified an interaction between STAT1 and CaMKII in TED-OFs, n = 3. **b**–**d** WB showed KN-93 phosphate pretreatment (10 μmol/L) for 1 h inhibited TGF-β1 induced p-CaMKII (Thr286/287) protein expression at 30 min and TGF-β1 induced p-STAT1(Ser727) protein expression at 2 h, in TED-OFs, n = 3. **e**–**h** WB showed fludarabine pretreatment (10 μmol/L) for 1 h suppressed TGF-β1 induced p-STAT1(Ser727) protein expression at 2 h and TGF-β1 induced α-SMA and Col1A1 protein expression at 48 h, in TED-OFs, n = 3. **i**–**l** WB showed that nimodipine pretreatment dose-dependently suppressed TGF-β1 induced p-STAT1 (Ser727) protein expression at 2 h and TGF-β1 induced α-SMA, Col1A1 protein expression at 48 h, in TED-OFs, n = 3. The significance was determined by unpaired Student’s t-test (**c**, **d**, **f**–**h**) or one-way ANOVA (**j**–**l**). *P < 0.05; **P < 0.01; ***P < 0.001; ns, not significant. CaMKII, Ca^2+^/calmodulin-dependent protein kinase II; STAT1, signal transducer and activator of transcription 1; OF, orbital fibroblast; Co-IP, co-immunoprecipitation; TED-OFs, OFs derived from patients with TED; WB, western blot analysis; TGF-β1, transforming growth factor-beta 1; α-SMA, alpha-smooth muscle actin; Col1A1, collagen type I alpha 1; GAPDH, glyceraldehyde-3-phosphate dehydrogenase; WCL, whole cell lysate; Flu, fludarabine; Nimo, nimodipine

Next, Co-IP confirmed the interaction of CaMKII and STAT1 in TED-OFs (Fig. 9a). KN-93 inhibited TGF-β1 induced phosphorylation of STAT1, suggesting that STAT1 is a downstream protein of CaMKII (Fig. 9b–d), consistent with previous findings [65, 66]. We next evaluated the effect of fludarabine, a specific inhibitor for STAT1 activation, in the TGF-β1 induced in vitro TED model. The results showed that fludarabine reduced the protein expression levels of α-SMA and Col1A1 induced by TGF-β1 (Fig. 9e–h). CCK-8 assays confirmed that concentrations of fludarabine below 20 μmol/L were safe for OFs (Additional file 6: Fig. S5). All these findings demonstrate that the activation of the CaMKII/STAT1 signaling pathway play an essential role in TGF-β1 mediated pro-fibrotic mechanisms in TED-OFs.

Additionally, analysis of transcriptional target genes based on RNA sequencing data unveiled an attenuation in STAT1 signaling subsequent to nimodipine pretreatment (Additional file 7: Fig. S6). Nimodipine pretreatment down-regulated TGF-β1 induced STAT1 phosphorylation level and reduced downstream α-SMA and Col1A1 protein expression levels in a dose-dependent manner (Fig. 9i–l). Taken together, these results provide evidence that nimodipine exerts anti-fibrotic effects by suppressing the CaMKII/STAT1 signaling pathway.

## Discussion

This study firstly illustrated the role of LTCC in mediating TGF-β1 induced pro-fibrotic mechanisms in TED. Additionally, we demonstrated that a well-tolerated LTCC inhibitor nimodipine delivered potent anti-fibrotic effects by reducing TGF-β1 induced Ca^2+^ response and downstream expression of pro-fibrotic genes and proteins in TED-OFs, as well as alleviating the TGF-β1 induced cell proliferation and OFs migration. Importantly, our data provided evidence that activation of the CaMKII/STAT1 signaling pathway participates in fibrogenesis during TED. Mechanistically, nimodipine exerted anti-fibrotic effects by suppressing the CaMKII/STAT1 signaling pathway.

To date, the demand for inhibiting orbital tissue fibrosis in TED remains unfulfilled, and further investigations into the mechanisms underlying fibrosis in TED are warranted [11, 13, 22]. A substantial amount of evidence has illustrated that myofibroblast transdifferentiation, proliferation and migration of OFs induced by TGF-β1 is the primary pathophysiological process in fibrogenesis during TED [11, 23, 55–58]. Therefore, further exploration of the TGF-β1 signaling pathway may hold potential for addressing these issues. Recently, Hou et al. suggested that c-Jun N-terminal kinase (JNK) and p38 pathways were involved in fibrogenesis during TED, and administration of JNK and p38 inhibitors attenuated TGF-β1 induced fibrogenesis in OFs [67]. However, those inhibitors have not yet been used in clinical practice. Recent investigations also suggested that curcumin and gypenosides can alleviate TGF-β1 induced myofibroblast transdifferentiation in OFs [68, 69]. However, the safety data for these drugs are insufficient, and their long-term use may lead to potential side effects. The latest studies have revealed the crucial involvement of Ca^2+^ signaling in the regulation of fibroblast function, with its mechanism intricately linked to TGF-β1 signaling transduction [30, 31, 51, 70]. LTCC has been demonstrated to be distinct and essential in mediating Ca^2+^ signal transduction in excitable cells [33]. Of note, recent work has revealed the role of LTCC in mediating Ca^2+^ response and critical downstream signaling events in T and B cells as well as lung fibrocytes [28, 71, 72]. Moreover, the administration of LTCC blockers has exhibited pronounced anti-fibrotic effects in preclinical investigations encompassing cardiovascular, pulmonary, hepatic, and urological disorders [27, 28, 73, 74]. Hence, it is imperative to investigate whether LTCC plays a role in the modulation of OFs functions.

Previous studies have showed that the LTCC complex consists of three subunits: (1) The α1 subunit serves as the crucial component of the LTCC by constituting a selective pore that facilitates the passage of Ca^2+^ ions, and hosting the majority of binding sites for regulatory proteins and drugs, particularly dihydropyridines (DHPs); (2) The auxiliary subunits α2δ and β2 are involved in the anchoring, transportation, and regulation of the LTCC complex [54, 75]. Based on previous research, we conducted mRNA transcriptome sequencing analysis to investigate the expression of genes encoding crucial LTCC subunits in TED-OFs. The results firstly revealed a high abundance of gene expression of Ca_v_1.2α1 (CACNA1C) in TED-OFs, and an extremely low abundance of gene expression of Ca_v_1.3α1, with no gene expression of Ca_v_1.1α1 and Ca_v_1.4α1, supporting previous studies [34, 54]. However, there was no significant difference in the expression of two other genes (CACNA2D1 and CACNB2) associated with auxiliary subunits Ca_v_α2δ and Ca_v_β2 of LTCC between the two groups, which have been reported to perform functions independent of the Ca^2+^ channel [75–81]. Importantly, the Ca_v_1.2α1 subunits possess all the key features that define a LTCC [54]. However, there is currently no literature defining the role of LTCC and its subunits in TED. Our study provides evidence of an up-regulation in the expression level of CACNA1C, which encodes Ca_v_1.2α1, in TED-OFs, compared to HC-OFs (Fig. 1a). The RNA sequencing data from GSE58331 also supports our findings by illustrating that CACNA1C was up-regulated in patients with TED. Moreover, our results suggested the presence of a functional LTCC that mediates differentiation, cellular proliferation and motility of TED-OFs and is involved in TGF-β1 induced pro-fibrotic mechanisms in TED. Hence, the application of LTCC blockers may be novel therapeutic strategies for fibrosis in TED.

CaMKII is a multifunctional serine/threonine kinase that is ubiquitously expressed throughout the body and known to be critical in regulating the Ca^2+^ signaling pathway [59]. When the intracellular Ca^2+^ concentration increases, the Ca^2+^/calmodulin complex binds to the corresponding CaMKII domain and activates the subunits of CaMKII by phosphorylation in the regulatory domain, which initiates the activation of CaMKII holoenzyme and serves as a vital modulator in downstream cascade [60, 82]. Since we had found that TGF-β1 induced Ca^2+^ response in TED-OFs, it is worthwhile to determine the role of CaMKII signaling in fibrogenesis during TED. In this study, we have demonstrated, for the first time, a significant increase in CaMKII phosphorylation in the orbital adipose connective tissues of patients with TED. Moreover, TGF-β1 induces the phosphorylation of CaMKII in TED-OFs, consistent with a previous finding in human pulmonary fibroblasts [83]. Furthermore, selective inhibition of CaMKII by a specific inhibitor, KN-93, attenuated the TGF-β1 induced pro-fibrotic functions of OFs, in line with previous studies investigating pulmonary fibrosis [83], ureteral scar formation [74], and adverse cardiac remodeling [84]. Our results offer evidence that the activation of CaMKII signaling plays a pivotal role in fibrogenesis during TED, and thus support the hypothesis that Ca^2+^ signaling actively contributes to the development of fibrosis in TED. These findings also suggest that CaMKII may be a promising therapeutic target for fibrosis in TED.

STAT1 is the first member of the STAT family and serves as a key modulator in a variety of cellular functions, including immune response, apoptosis, cell growth and differentiation [85]. Recent studies report that the activation of STAT1 signaling plays a crucial role in chronic liver fibrosis [86, 87] and can be induced by TGF-β1 in vitro [88]. Furthermore, α-SMA has been demonstrated to be a downstream protein of STAT1 [89], and inhibition of STAT1 signaling ameliorates tubulointerstitial fibrosis in diabetic kidney disease [63], attenuates pulmonary vascular fibrosis [62], and rescues the exacerbated remodeling in myocardial infarction [64]. Besides, STAT1 has been shown to be activated by CaMKII [65, 66]. Recent studies also revealed that statins protect against the development of TED and alleviate orbital fibrosis by their pleiotropic effects [90–92]. Interestingly, statins have been reported to inhibit LTCC activity and STAT1-mediated gene transcription [93–95]. Therefore, we assume that statins may deliver their therapeutic effects in TED by targeting LTCC and STAT1. Therefore, it is imperative to investigate the potential involvement of STAT1 in fibrogenesis during TED. Importantly, our study first revealed an increased STAT1 phosphorylation level both in the orbital adipose connective tissues of patients with TED and in TED-OFs. Furthermore, WB identified that TGF-β1 induced STAT1 phosphorylation in TED-OFs, and inhibition of the STAT1 signaling pathway by fludarabine abolished the TGF-β1 induced expression of fibrotic proteins, in line with previous findings [62, 63]. Additionally, Co-IP and WB verified that STAT1 was a downstream protein of CaMKII, consistent with previous reports [65, 66]. Collectively, we conclude that activation of the CaMKII/STAT1 signaling pathway participates in the fibrogenesis process during TED. STAT1 may be a potential therapeutic target for the management of fibrosis in TED as well.

Nimodipine, a highly selective dihydropyridine LTCC blocker, has received approval from the U.S. Food and Drug Administration (FDA) for the prevention and treatment of neurological deficits in patients suffering from aneurysmal subarachnoid hemorrhage (aSAH) [39]. In past decades, nimodipine was initially thought to deliver its effect by relaxing cerebral vascular smooth muscle and attenuating the ischemic consequences of angiographic vasospasm [96, 97]. However, pivotal studies have demonstrated contradictory results in that the administration of nimodipine did not yield a significant impact on angiographic vasospasm, yet the clinical outcomes were improved [98, 99]. From then on, the neuroprotection effects of nimodipine have been widely studied, and its intricate mechanisms in a myriad of cell types have been demonstrated [100–104]. Due to its excellent lipophilic property, it can easily penetrate the blood–brain barrier, so early studies focused on brain diseases [39]. Recent work in normal tension glaucoma (NTG) reported that oral administration of nimodipine not only distributed well in ocular circulation, but also improved the contrast sensitivity of color vision significantly [41, 42]. Preclinical studies in multiple sclerosis, autoimmune encephalomyelitis and autoimmune uveitis also suggested that nimodipine has potential immunomodulatory effects by inhibiting the release of inflammatory factors by microglia cells and maintaining the balance of effector T cells/regulatory T cells [44–46]. These advantages make it an excellent potential treatment candidate for fibrosis in TED, as the orbit is full filled with fat, and immune cells infiltrating in the orbit promotes the fibrosis progression of TED [5, 8]. Our study provides a theoretical basis for nimodipine as a potential alternative agent for fibrosis in TED. Due to the vast cost and long period in developing new drugs, conventional drugs with novel uses are greatly cost-effective. Additionally, the potential mild anti-hypertensive and neuroprotective effects of nimodipine may confer benefits on patients with TED who have comorbid cardiovascular and cerebrovascular conditions, particularly those experiencing post-glucocorticoid therapy hypertension. Based on the above, nimodipine may be a potential candidate for treating TED.

Our study has some limitations. We failed to evaluate the anti-fibrotic effects and optimal doses of nimodipine in vivo due to the current unavailability of applicable and stable animal models for TED. Furthermore, the therapeutic effects and mechanisms of nimodipine in TED are still worth exploring for potential clinical use, and the biological functions and subcellular localization of LTCC in OFs still require further investigations. Current research indicates a crucial role of CD4+ T cell subset Th17 cells in fibrogenesis during TED by actively interacting with OFs and promoting transdifferentiation of myofibroblasts induced by TGF-β1 while inhibiting adipogenesis in OFs through their secretion of cytokine IL-17A [11, 13, 22]. In this study, we mainly focused on the downstream effects of TGF-β1, the modulation of the production of TGF-β1 is also a focal point for our future research.

## Conclusions

Our study demonstrates that TGF-β1 induces an LTCC-mediated Ca^2+^ response, followed by activation of the CaMKII/STAT1 signaling pathway, which is involved in fibrogenesis during TED. Nimodipine, a LTCC blocker, exerts potent anti-fibrotic effects in the TGF-β1 induced in vitro TED model by suppressing the CaMKII/STAT1 signaling pathway. Our results deepen the understanding of fibrogenesis during TED, provide novel therapeutic targets, and shed some light on future research directions for the management of fibrosis in TED.

## Supplementary Information

**Additional file 1: Table S1.** Genes associated with vital subunits of LTCC in TED-OFs.

**Additional file 2: Fig. S1.** Heatmap showing the up-regulation of CACNA1C in patients with thyroid eye disease (TED).

**Additional file 3: Fig. S2.** Cytotoxicity test of nimodipine in OFs. TED-OFs were treated with 20–100 μmol/L nimodipine for 24 or 48 h. Cell viability was assessed using the CCK-8 assay, n = 3, two-way ANOVA. Every concentration at different time points showed no statistically significant difference when compared with the control. OF, orbital fibroblast; TED-OFs, OFs derived from patients with thyroid eye disease; CCK-8, cell counting kit-8.

**Additional file 4: Fig. S3.** Nimodipine attenuated TGF-β1 induced cell proliferation of OFs. **a–b** Representative images and statistical analyses of EdU-positive TED-OFs in different groups detected by flow cytometry. Before EdU assay, OFs were pretreated with 0 (control), 20, 40 or 60 μmol/L nimodipine for 5 min, followed by 10 ng/mL TGF-β1 stimulation for 24 h, n = 4. ****P < 0.0001, one-way ANOVA. TGF-β1, transforming growth factor-beta 1; OF, orbital fibroblast; EdU, 5-ethynyl-2′-deoxyuridine proliferation assay; TED-OFs, OFs derived from patients with thyroid eye disease; Nimo, nimodipine.

**Additional file 5: Fig. S4.** Cytotoxicity test of KN-93 in OFs. TED-OFs were treated with 5–40 μmol/L KN-93 for 24 or 48 h. Cell viability was assessed using the CCK-8 assay, n = 3. Only the 40 μmol/L KN-93 treatment decreased cell viability at 48 h (*P < 0.05, compared to the control group, two-way ANOVA). The other concentrations at different time points showed no significant differences when compared with the control. KN-93, KN-93 phosphate; OF, orbital fibroblast; TED-OFs, OFs derived from patients with thyroid eye disease; CCK-8, cell counting kit-8.

**Additional file 6: Fig. S5.** Cytotoxicity test of fludarabine in OFs. TED-OFs were treated with 5–40 μmol/L fludarabine for 24 or 48 h. Cell viability was assessed using the CCK-8 assay, n = 3. Only the 40 μmol/L fludarabine treatment decreased cell viability at 48 h (*P < 0.05, compared to the control group, two-way ANOVA). The other concentrations at different time points showed no significant differences when compared with the control. OF, orbital fibroblast; TED-OFs, OFs derived from patients with thyroid eye disease; CCK-8, cell counting kit-8.

**Additional file 7: Fig. S6.** Nimodipine exerts anti-fibrotic effects by suppressing the STAT1 signaling pathway. The potential transcriptional factor underlying the effect of nimodipine as well as the target genes were explored by the transcriptional target gene analysis. The heatmap exhibited a significant down-regulation of target genes associated with the STAT1 signaling pathway after nimodipine pretreatment (n = 3, each group). STAT1, signal transducer and activator of transcription 1; TGF-β1, transforming growth factor-beta 1; Nimo, nimodipine.

## Data Availability

RNA sequencing data has been deposited at the Genome Sequence Archive under the access code HRA006469. Other data in this study are included in the article.

## Abbreviations

α-SMA: Alpha-smooth muscle actin
Col1A1: Collagen type I alpha 1
Col1A2: Collagen type I alpha 2
CaMKII: Ca^2+^/calmodulin-dependent protein kinase II
Co-IP: Co-immunoprecipitation assay
DMEM/F12: A mixture of Dulbecco’ Modified Eagle Medium/Ham’s Nutrient Mixture F-12 (1:1 ratio)
EdU: 5-Ethynyl-2′-deoxyuridine proliferation assay
FBS: Fetal bovine serum
FCM: Flow cytometry
Flu: Fludarabine
FMO: Fluorescence minus one
HC: Healthy control
HC-OFs: OFs derived from HC donors
IHC: Immunohistochemistry
KN-93: KN-93 phosphate
LTCC: L-type calcium channel
Nimo: Nimodipine
OF: Orbital fibroblast
RT-qPCR: Real-time quantitative polymerase chain reaction
STAT1: Signal transducer and activator of transcription 1
TED: Thyroid eye disease
TGF-β1: Transforming growth factor-beta 1
TED-OFs: OFs derived from patients with TED
WB: Western blot analysis
WCL: Whole cell lysate
