# Supplementary material for: An L-type calcium channel blocker nimodipine exerts anti-fibrotic effects by attenuating TGF-β1 induced calcium response in an in vitro model of thyroid eye disease
Source: Eye Vis (Lond). 2024 Sep 6;11:37. doi: 10.1186/s40662-024-00401-5 (PMC11378575; doi:10.1186/s40662-024-00401-5)
Supplement: Supplementary file 1 — Additional file 1: Table S1. Genes associated with vital subunits of LTCC in TED-OFs. [file 40662_2024_401_MOESM1_ESM.docx]

**Table S1.** Genes associated with vital subunits of LTCC in TED-OFs

| Subunits | Genes | Expression (mean±SD) |
| --- | --- | --- |
| Ca_V_1.1α1 | CACNA1S | 0 |
| Ca_V_1.2α1 | CACNA1C | 408.667±230.099 |
| Ca_V_1.3α1 | CACNA1D | 4.333±4.041 |
| Ca_V_1.4α1 | CACNA1F | 0 |
| Ca_V_α2δ-1 | CACNA2D1 | 916.333±284.662 |
| Ca_V_α2δ-2 | CACNA2D2 | 2.333±2.517 |
| Ca_V_α2δ-3 | CACNA2D3 | 7.000±6.000 |
| Ca_V_α2δ-4 | CACNA2D4 | 9.667±7.024 |
| Ca_V_β2 | CACNB2 | 14.000±4.583 |

LTCC = L-type calcium channel; TED-OFs = OFs derived from patients with TED; SD = standard deviation.
