# Supplementary material for: An L-type calcium channel blocker nimodipine exerts anti-fibrotic effects by attenuating TGF-β1 induced calcium response in an in vitro model of thyroid eye disease
Source: Eye Vis (Lond). 2024 Sep 6;11:37. doi: 10.1186/s40662-024-00401-5 (PMC11378575; doi:10.1186/s40662-024-00401-5)

Bulk RNA-seq data from GSE58331

Comparison of anterior orbit tissues from healthy donors versus patients with TED

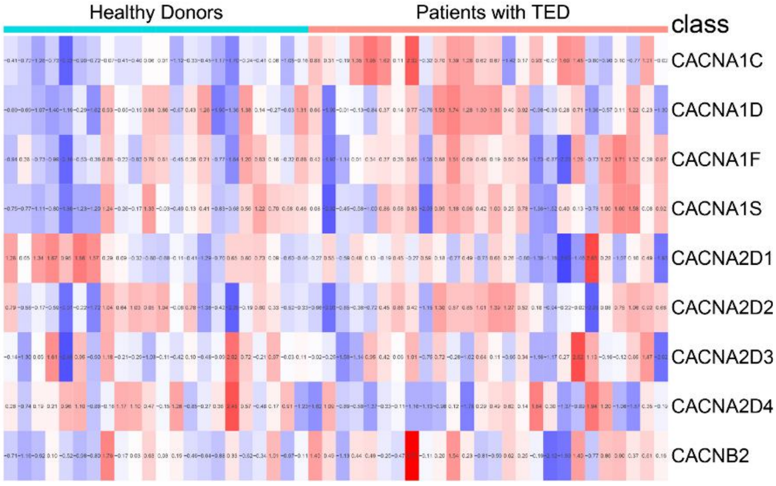

class	log2FC	adj.P.Val
CACNA1C	0.758462	1.74E-5
CACNA1D	-0.28265	0.204354
CACNA1F	-0.30408	0.204354
CACNA1S	0.205899	0.300556
CACNA2D1	0.16493	0.340004
CACNA2D2	0.173283	0.372373
CACNA2D3	0.178618	0.372373
CACNA2D4	0.141701	0.372373
CACNB2	-0.00094	0.993167

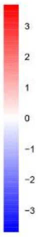

Supplement: Supplementary file 2 — Additional file 2: Fig. S1. Heatmap showing the up-regulation of CACNA1C in patients with thyroid eye disease (TED). [file 40662_2024_401_MOESM2_ESM.pdf]
