# Supplementary figures and images for: An L-type calcium channel blocker nimodipine exerts anti-fibrotic effects by attenuating TGF-β1 induced calcium response in an in vitro model of thyroid eye disease
Source: Eye Vis (Lond). 2024 Sep 6;11:37. doi: 10.1186/s40662-024-00401-5 (PMC11378575; doi:10.1186/s40662-024-00401-5)

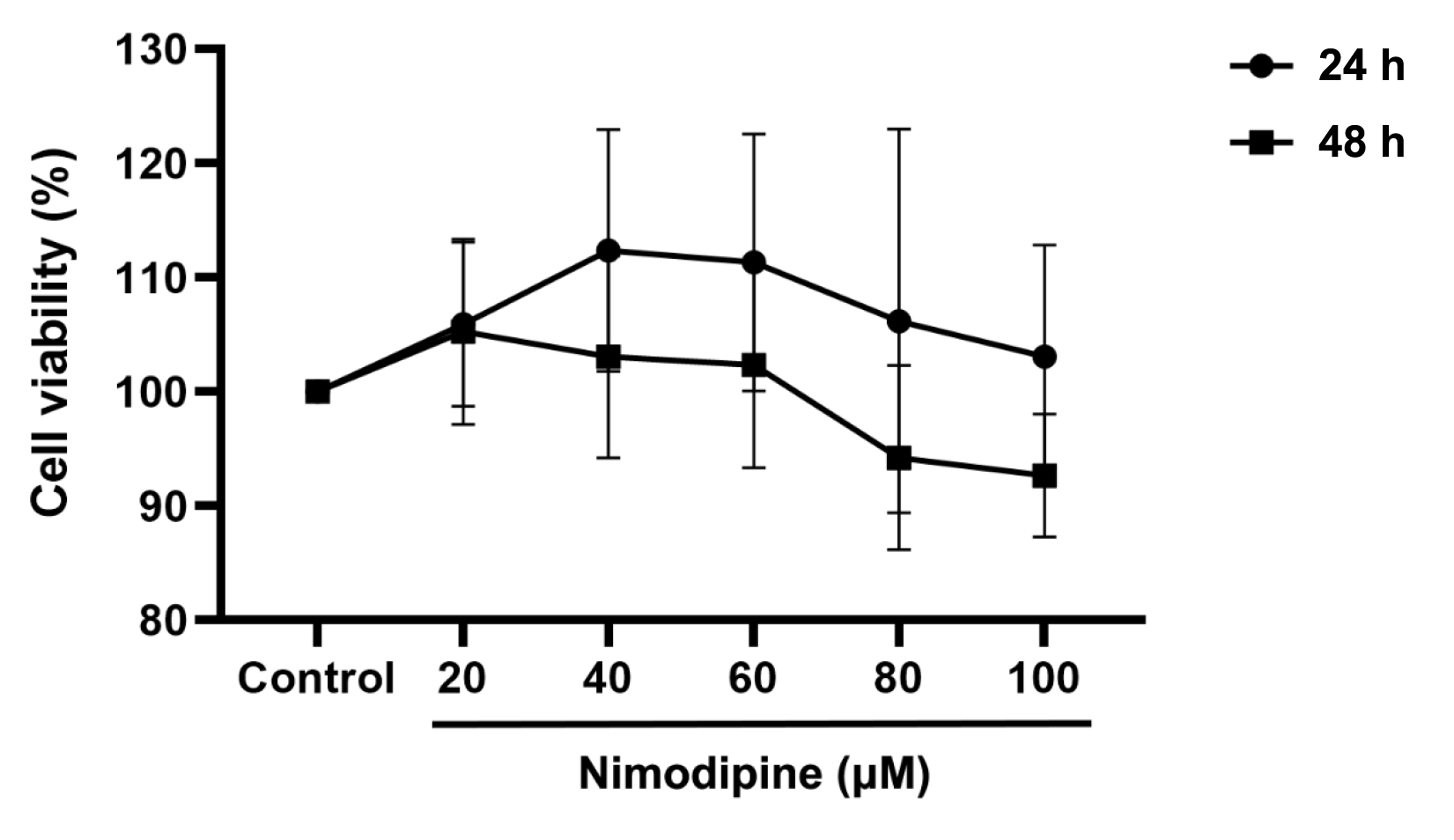

Supplement: Supplementary file 3 — Additional file 3: Fig. S2. Cytotoxicity test of nimodipine in OFs. TED-OFs were treated with 20–100 μmol/L nimodipine for 24 or 48 h. Cell viability was assessed using the CCK-8 assay, n = 3, two-way ANOVA. Every concentration at different time points showed no statistically significant difference when compared with the control. OF, orbital fibroblast; TED-OFs, OFs derived from patients with thyroid eye disease; CCK-8, cell counting kit-8. [file 40662_2024_401_MOESM3_ESM.tif]

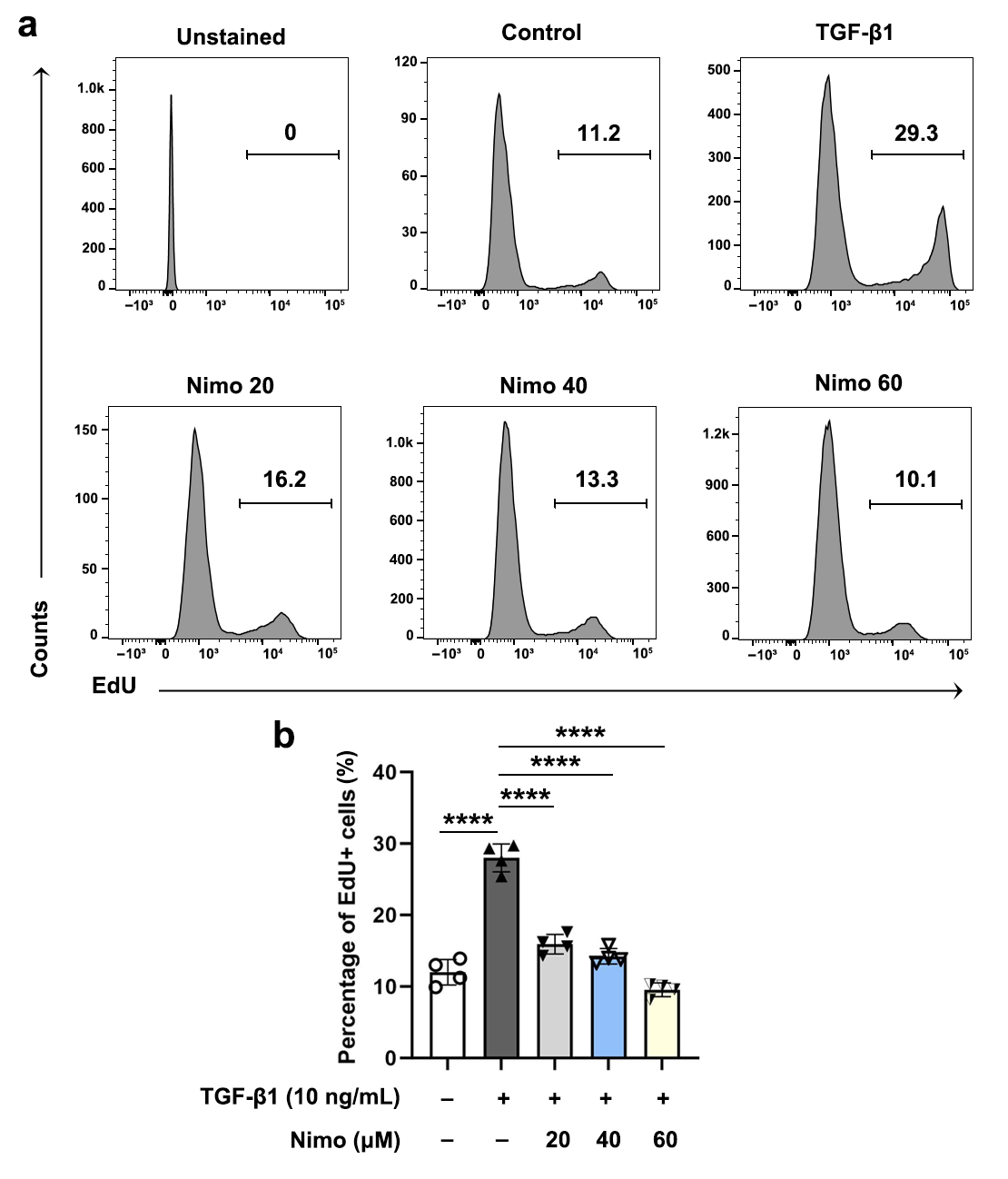

Supplement: Supplementary file 4 — Additional file 4: Fig. S3. Nimodipine attenuated TGF-β1 induced cell proliferation of OFs. a–b Representative images and statistical analyses of EdU-positive TED-OFs in different groups detected by flow cytometry. Before EdU assay, OFs were pretreated with 0 (control), 20, 40 or 60 μmol/L nimodipine for 5 min, followed by 10 ng/mL TGF-β1 stimulation for 24 h, n = 4. ****P < 0.0001, one-way ANOVA. TGF-β1, transforming growth factor-beta 1; OF, orbital fibroblast; EdU, 5-ethynyl-2′-deoxyuridine proliferation assay; TED-OFs, OFs derived from patients with thyroid eye disease; Nimo, nimodipine. [file 40662_2024_401_MOESM4_ESM.tif]

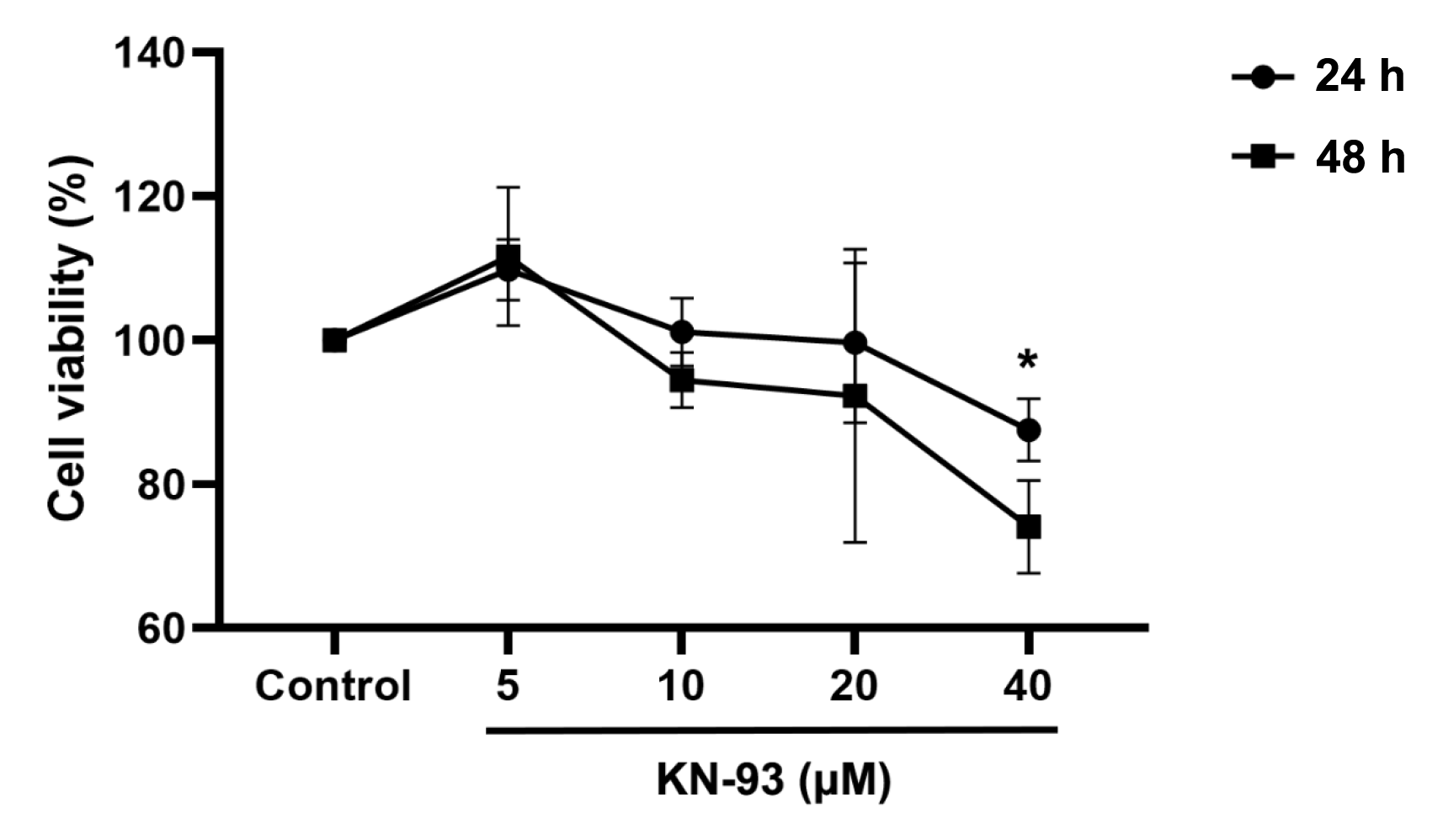

Supplement: Supplementary file 5 — Additional file 5: Fig. S4. Cytotoxicity test of KN-93 in OFs. TED-OFs were treated with 5–40 μmol/L KN-93 for 24 or 48 h. Cell viability was assessed using the CCK-8 assay, n = 3. Only the 40 μmol/L KN-93 treatment decreased cell viability at 48 h (*P < 0.05, compared to the control group, two-way ANOVA). The other concentrations at different time points showed no significant differences when compared with the control. KN-93, KN-93 phosphate; OF, orbital fibroblast; TED-OFs, OFs derived from patients with thyroid eye disease; CCK-8, cell counting kit-8. [file 40662_2024_401_MOESM5_ESM.tif]

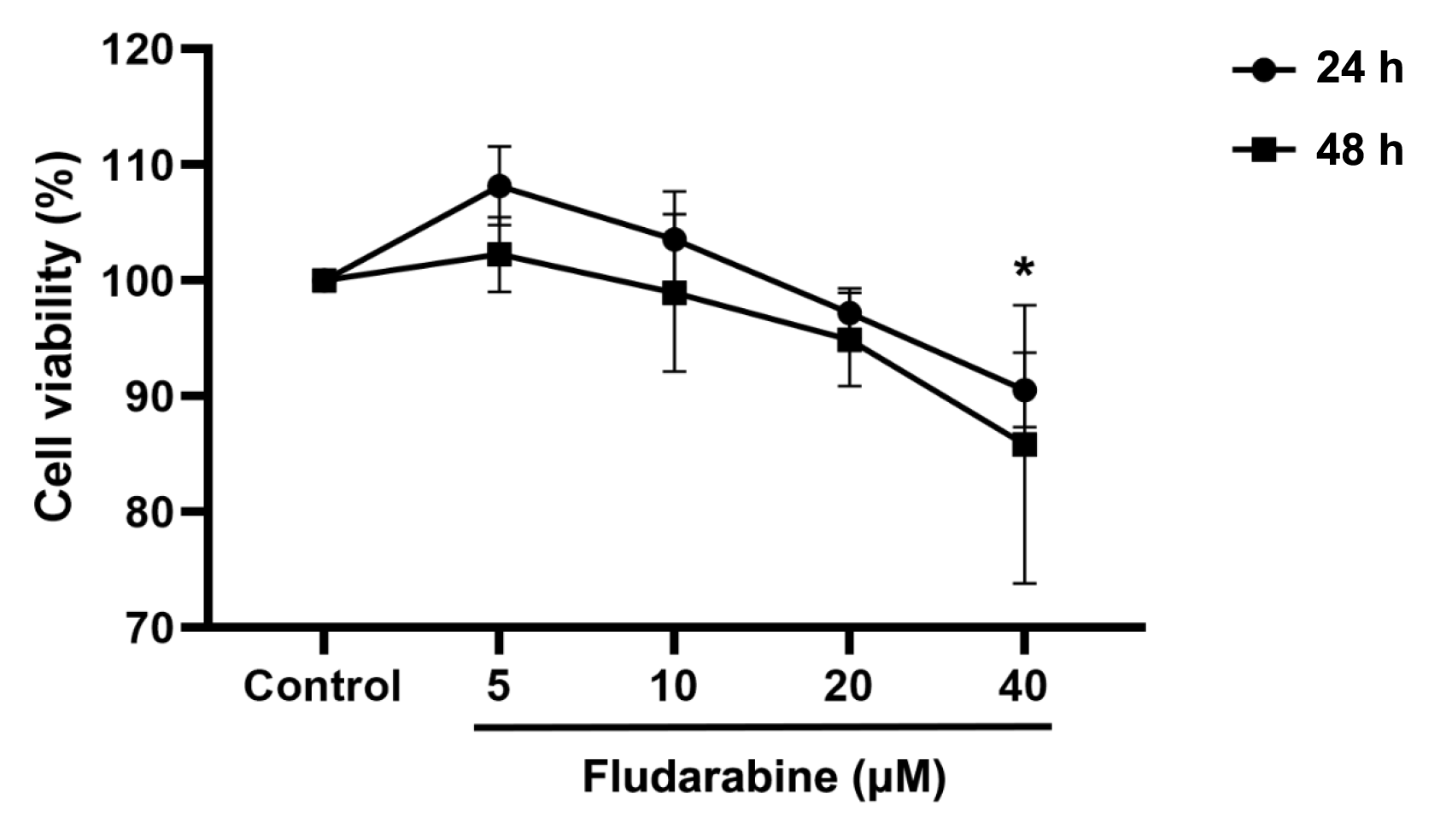

Supplement: Supplementary file 6 — Additional file 6: Fig. S5. Cytotoxicity test of fludarabine in OFs. TED-OFs were treated with 5–40 μmol/L fludarabine for 24 or 48 h. Cell viability was assessed using the CCK-8 assay, n = 3. Only the 40 μmol/L fludarabine treatment decreased cell viability at 48 h (*P < 0.05, compared to the control group, two-way ANOVA). The other concentrations at different time points showed no significant differences when compared with the control. OF, orbital fibroblast; TED-OFs, OFs derived from patients with thyroid eye disease; CCK-8, cell counting kit-8. [file 40662_2024_401_MOESM6_ESM.tif]

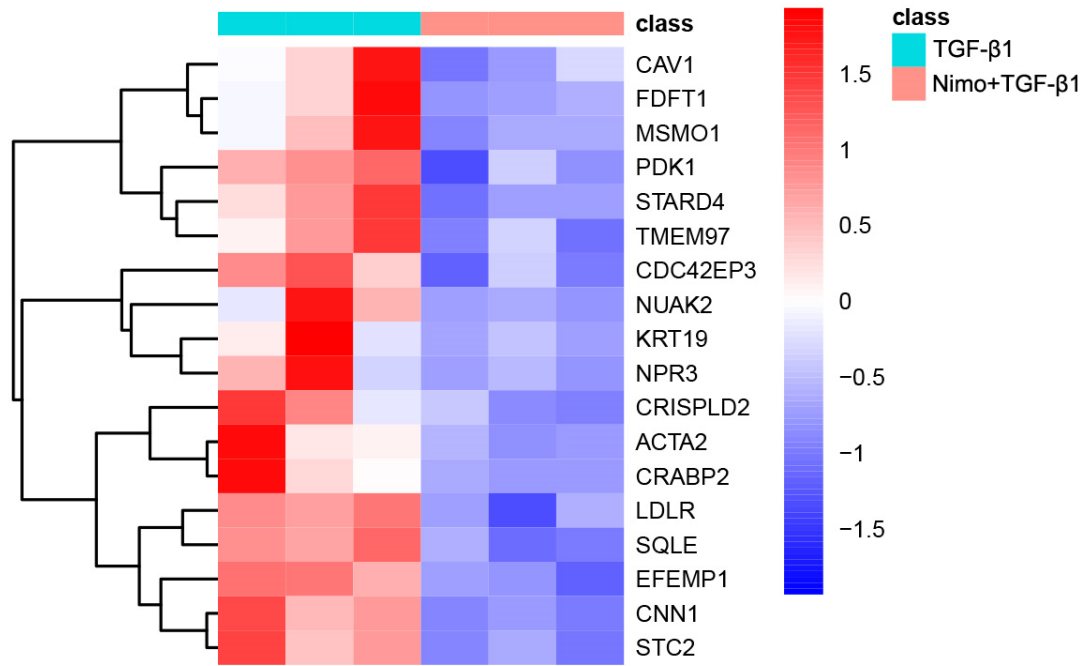

Supplement: Supplementary file 7 — Additional file 7: Fig. S6. Nimodipine exerts anti-fibrotic effects by suppressing the STAT1 signaling pathway. The potential transcriptional factor underlying the effect of nimodipine as well as the target genes were explored by the transcriptional target gene analysis. The heatmap exhibited a significant down-regulation of target genes associated with the STAT1 signaling pathway after nimodipine pretreatment (n = 3, each group). STAT1, signal transducer and activator of transcription 1; TGF-β1, transforming growth factor-beta 1; Nimo, nimodipine. [file 40662_2024_401_MOESM7_ESM.pdf]
